# The Efficiency and Cost-Effectiveness of Wearable Sensors in a Digital Physiotherapeutic Total Hip Arthroplasty–Specific Training System for Patients After Total Hip Arthroplasty: Randomized Controlled Trial

**DOI:** 10.2196/93050

**Published:** 2026-07-21

**Authors:** Chenyi Jiang, Yun Shen, Lihua Huang, Yanhong Ma, Shengdi Lu, Jimin Yin

**Affiliations:** 1 Department of Orthopaedics, Shanghai Sixth People's Hospital, 600 Yishan Road, Shanghai, 200233, China, 1 6265540472; 2 Pennington Biomedical Research Center, 6400 Perkins Rd, Baton Rouge, LA, 70808, United States; 3 Department of Rehabilitation Medicine, Shanghai Sixth People's Hospital, Shanghai, China

**Keywords:** total hip arthroplasty, wearable sensors, telerehabilitation, cost-effectiveness, patient adherence

## Abstract

**Background:**

With total hip arthroplasty (THA) volumes rising worldwide, scalable home rehabilitation strategies are needed. Home-based digital physiotherapy can improve recovery after THA, and adding wearable motion sensor feedback may further enhance exercise performance and adherence. However, the impact of sensor-augmented digital rehabilitation on patient outcomes and cost-effectiveness remains unclear.

**Objective:**

This study aimed to evaluate the clinical effectiveness (including adherence) and cost-effectiveness of adding wearable sensor feedback to a home-based digital THA rehabilitation program compared with the same digital program without sensors.

**Methods:**

We conducted a single-center randomized controlled trial in Shanghai from June 2023 to June 2024. A total of 240 patients who had undergone primary THA were randomized (1:1) to a 12-week home exercise program delivered via a mobile app either with wearable motion sensors for real-time feedback (intervention) or without sensors (control). Both groups received identical exercise content and weekly teleconsultations. Outcomes were assessed at 6, 12, and 24 weeks by blinded evaluators. The primary outcome was the Hip Disability and Osteoarthritis Outcome Score (HOOS) at 24 weeks. Secondary outcomes included HOOS subscales, timed up-and-go (TUG), Berg Balance Scale, 36-item Short Form Health Survey (SF-36) physical and mental scores, Hospital Anxiety and Depression Scale (HADS) anxiety/depression, patient satisfaction, adherence metrics, and total 24-week costs. Intention-to-treat analyses were used. Mixed effects models and *χ*² tests were used for group comparisons. Cost-effectiveness was evaluated from a societal perspective.

**Results:**

The sensor-based rehabilitation group showed significantly greater improvement in the primary outcome (HOOS overall) than the control group at 24 weeks (*P*=.01). Early postoperative gains were larger: At 6 weeks, 4 of the 5 HOOS subscales, TUG time, and all patient-reported outcomes remained significant after Bonferroni correction for 47 secondary comparisons (adjusted *P*<.001). By 24 weeks, hip-specific functional differences had narrowed, but SF-36 physical and mental scores (both *P*<.001) and HADS anxiety and depression scores (both *P*<.001) remained significant after correction. Total 24-week costs were similar between the groups (¥87,967 vs ¥94,396 [US $12,879.80 vs $13,821.10] per patient; *P*=.32). The sensor intervention was associated with numerically lower costs on average (¥6428.98 [US $941.31] less per patient), yielding negative incremental cost-effectiveness ratios, though the cost difference was not statistically significant. Adherence was high in both groups but higher with sensors: The intervention group completed more exercise sessions (*P*=.002) and showed greater participation in weekly assessments and follow-up calls (both *P*<.001). Adverse events were uncommon in both groups (5.8% vs 7.5%) and mostly minor, with no serious events reported.

**Conclusions:**

Augmenting home-based digital rehabilitation with wearable sensor feedback led to statistically significant improvements in the primary hip function outcome and in patient-reported quality of life and psychological outcomes that persisted after Bonferroni correction through 24 weeks. Several early hip-specific secondary outcome differences did not survive correction for multiple comparisons. The between-group HOOS differences were below the established minimal clinically important difference. Exercise adherence was significantly higher in the sensor group. These benefits were achieved with no increase in cost, suggesting the sensor-enhanced program is a safe and feasible approach to THA rehabilitation that enhances adherence and accelerates early recovery.

## Introduction

Total hip arthroplasty (THA) is in increasingly high demand worldwide driven by aging populations and expanding indications. Over 1 million THAs are performed annually, and procedural volumes are projected to rise dramatically in coming decades (an estimated 174% increase in primary THA by 2030 in the United States) [1]. As the number of THA surgeries grows, there is a critical need for scalable and accessible postoperative rehabilitation strategies that can accommodate larger patient volumes without compromising outcomes. Effective rehabilitation is essential to maximize functional recovery after THA and to ensure long-term success of the procedure. However, traditional clinic-based physiotherapy can be resource-intensive and may pose accessibility challenges, especially for patients with mobility limitations or those living far from specialized centers.

Digital health interventions and home-based rehabilitation programs have emerged as promising solutions to meet this need. Recent evidence supports the efficacy of telerehabilitation and other digital home exercise programs following joint replacement. Randomized trials have shown that home-based digital rehabilitation yields postoperative functional outcomes comparable to those of conventional in-person therapy [2,3]. Even in post-THA telerehabilitation without specialized sensors, compliance with home exercise has been observed to improve relative to standard care [3]. Moreover, a digital program has demonstrated superior short-term results: An artificial intelligence–powered home exercise system with biofeedback after THA led to significantly greater gains in mobility and higher patient-reported outcome scores than traditional rehabilitation in a pilot trial [2]. These trials underscore that technology-assisted, home-based rehabilitation can expand access without sacrificing effectiveness and, in certain cases, may enhance early recovery metrics.

Wearable sensor technology offers an innovative enhancement to digital rehabilitation. By integrating inertial motion sensors into home exercise regimens, patients can receive real-time biofeedback on their exercise technique and performance. This immediate feedback loop is posited to improve the quality of exercise execution and boost patient engagement and adherence. Early data support these benefits: Sensor-based telerehabilitation systems have been shown to increase patient adherence and exercise intensity during orthopedic recovery [4,5]. In one study of a knee rehabilitation app, patients using wearable motion sensors achieved higher rates of prescribed exercise completion and greater training intensity than those undergoing usual care, with no safety concerns [5]. These observations suggest that wearable sensor feedback could further amplify the positive effects of home rehabilitation by motivating patients and ensuring exercises are performed correctly, which in turn may translate to better functional outcomes.

Beyond clinical efficacy, home-based digital rehabilitation has important health economic implications. Remote programs can reduce the burden of travel and time costs for patients and caregivers, increasing the convenience and uptake of rehabilitation. From a health system perspective, shifting rehabilitation to the home setting may help contain costs and resource utilization [6]. This reduced “time burden” with telerehabilitation reflects potential indirect cost savings (such as fewer work absences and transportation expenses) and supports digital rehab as a cost-effective, patient-centered alternative to facility-based care [6]. Additionally, decreasing reliance on in-person supervision through digital tools could alleviate workforce demands and enable broader dissemination of rehab services to underserved or remote regions [2-6].

Building on this background, this randomized controlled trial (RCT) was designed to evaluate the added value of wearable sensor feedback in home-based digital rehabilitation following THA. We hypothesized that integrating wearable sensor–mediated biofeedback into a home-based digital rehabilitation program would improve patient adherence and lead to superior recovery after THA compared with a digital rehabilitation program without sensor feedback.

## Methods

### Study Design and Setting

This study was a single-center, 2-arm, parallel-group RCT reported in accordance with the CONSORT (Consolidated Standards of Reporting Trials) 2010 guidelines. It was conducted at the Chinese National Medical Center of Orthopaedics in Shanghai, China (affiliated with Shanghai Sixth People’s Hospital) from June 2023 to June 2024.

Eligible patients awaiting primary THA at the study center were identified from surgical waiting lists and screened by two physicians according to predefined inclusion criteria. Patients meeting eligibility were enrolled prior to surgery. Baseline (preoperative) evaluations for all participants were performed by a single physiotherapist after consent. On the first postoperative day, participants were randomly allocated in a 1:1 ratio to the study groups by a trial coordinator (allocation details and sequence described later). Two independent physiotherapists (not involved in initial enrollment nor outcome assessment) subsequently contacted each participant to implement the allocated rehabilitation intervention. All participants then underwent a 12-week home-based rehabilitation program according to their group assignment and were followed for a total of 24 weeks postsurgery. The 24-week follow-up duration was selected to capture both the acute rehabilitation phase, during which sensor-mediated benefits were expected to be most pronounced, and the later recovery period, during which convergence of outcomes was anticipated based on prior literature in musculoskeletal rehabilitation. Outcome assessments were conducted at baseline (presurgery) and at 6, 12, and 24 weeks after surgery by two physiotherapists in the outpatient clinic who were blinded to group allocation to minimize observer bias. These outcome evaluators, who were not involved in providing the interventions, collected all functional performance measures and assisted participants with completing self-reported outcome questionnaires using an online system during the clinic visits. Participant adherence to the digital training intervention was monitored through system usage logs; if a physiotherapist noted that a patient was not engaging with the digital rehabilitation platform as prescribed, they promptly inquired about the reasons for nonadherence to address any barriers to participation.

### Ethical Considerations

Ethics approval for this study was obtained from the Ethics Committee of Shanghai Sixth People’s Hospital (approval number: 2016KY-109(K)), and the study was conducted in accordance with institutional guidelines and international ethical standards, including the principles of the Declaration of Helsinki. Written informed consent was obtained from all participants prior to enrollment, and participation was voluntary. All data were anonymized to protect participants’ privacy and maintain confidentiality, ensuring that no personal identifiers were included in the analytical dataset. No compensation was provided to participants.

### Participants

This single-center trial enrolled patients scheduled for primary THA from the waiting list of the orthopedic surgery outpatient clinic of a tertiary hospital. Eligible patients were identified and consented preoperatively, and baseline assessments were completed before surgery. Potential participants were informed about the study 2 weeks prior to their admission and formally screened for eligibility on the same day of their admission. Those meeting all inclusion criteria were invited to participate, and written informed consent was obtained before enrollment.

The inclusion and exclusion criteria are shown in Textbox 1. Patients meeting the criteria were enrolled by the research coordinator and randomized to begin the assigned home-based rehabilitation program. All participants were recruited from a single high-volume arthroplasty center, ensuring a consistent surgical protocol and postoperative care across the study cohort. Each participant’s eligibility was verified against the criteria prior to randomization, and a screening log was maintained to record any exclusions or refusals.

Textbox 1. **Eligibility criteria.**

**Inclusion criteria**

- Age: 18 years to 80 years
- Underwent a primary THA within the prior 3 weeks for a nontraumatic indication (eg, osteoarthritis or osteonecrosis)
- Able to ambulate at least 10 meters (with or without a cane or walker) by approximately 2 weeks postsurgery
- Medically cleared by the surgical team for routine rehabilitation (no wound complications and no contraindications to unsupervised exercise, such as uncontrolled cardiac issues)
- Access to a smartphone or tablet with home internet to use the digital rehabilitation app (basic tech literacy or caregiver assistance as needed)
- Willing to be randomized and to comply with study procedures (including wearing the sensor device if assigned) and able to provide written informed consent

**Exclusion Criteria**

- Underwent a revision hip arthroplasty or any concurrent complex hip procedure during the index surgery (eg, periacetabular osteotomy or extensive muscle repair)
- Simultaneous bilateral THA in the same hospitalization
- Severe coexisting hip or lower-limb conditions that would impede rehabilitation (such as advanced arthritis in the opposite leg or significant neurological deficits affecting mobility/balance)
- Cognitive impairment or severe psychiatric illness that would prevent adherence to the exercise program or use of the technology
- Serious postoperative complications before enrollment that precluded normal rehab (eg, unstable medical status, a surgical site infection requiring intervention, or an unplanned reoperation for complications like dislocation)
- Already engaged in formal outpatient physiotherapy prior to enrollment that they were not willing to discontinue (for example, having started or completed an intensive supervised rehab program in the first 2 weeks post-THA)
- Concurrent participation in another interventional trial or prior participation in this study (such as a pilot enrollment)

### Intervention

All participants in both the intervention and control groups engaged in a 12-week home-based rehabilitation exercise program following THA. On the second postoperative day, each patient received an in-person orientation: One of the technicians assisted them with installing a dedicated mobile app (Joymotion software, Shanghai Medmotion Medical Management Co, Ltd) on their smartphone and provided instructions on its use. This app-based platform delivered a “Digital Physiotherapeutic THA-Specific Training System,” following the standardized post-THA training protocol (see Multimedia Appendix 1), and it recorded each patient’s exercise performance and completion rates. The supervising physiotherapist prescribed individualized daily exercise tasks through the app and could adjust the program remotely as needed. Both groups were scheduled for weekly teleconsultation sessions; at an appointed time each week, a physiotherapist from the rehabilitation center initiated a 2-way video call via the app to monitor progress, provide coaching, and address any issues. Notably, the frequency and duration of physiotherapist contact (including teleconsultations) were identical for the two groups to ensure parity in clinical guidance and support.

### Intervention Group

Participants allocated to the inertial measurement unit (IMU) intervention group received the home-based digital exercise program via the smartphone app with full telerehabilitation features. The app provided daily interactive exercise instructions, feedback on training performance, and real-time video or audio communication with the physiotherapist. The rehabilitation regimen was automatically tailored to each patient’s baseline characteristics and functional status, and the physiotherapist could further fine-tune the difficulty or exercises through a clinician interface. In addition to the app-based program, this group also used a wearable inertial sensor system to enhance exercise monitoring and feedback. Five small wireless IMU sensors were affixed to the patient’s body (one at the waist and one on the front of each thigh and each lower leg) and were registered and calibrated with the app during the initial setup. During each training session, these sensors captured the patient’s movements and projected them onto a virtual avatar visible in the app. The system’s software continuously compared the patient’s avatar movements with the pre-set correct posture for each exercise. If a movement did not reach the target position or was performed with improper form, the patient received immediate feedback from the app, prompting them to repeat the motion correctly. This real-time feedback mechanism ensured that patients in the IMU group could correct their technique on the spot, helping them perform the exercises as intended. Throughout the 12-week period, the assigned physiotherapists remotely monitored the IMU group’s exercise sessions via the app’s data logs. The sensor system transmitted detailed performance data to the therapist interface, allowing the physiotherapist to review each patient’s adherence, exercise form, and progress. Any necessary modifications to the exercise program or additional guidance were provided based on these observations.

### Control Group

Participants in the control group followed the same 12-week home exercise program using the digital platform but without the aid of wearable sensors. Like the IMU group, control group patients accessed daily THA-specific exercises and instructional videos through the mobile app, which tracked their exercise completion and provided basic performance feedback such as summaries of repetitions completed or time spent exercising. They received the same schedule of weekly physiotherapist-led teleconference sessions via the app’s two-way video interface, ensuring equal contact time and guidance as the intervention group. The exercise content and progression for control participants were also individualized to their baseline function and could be adjusted remotely by the physiotherapist in the same manner as for the IMU group. However, unlike the IMU group, the control group did not receive real-time motion feedback on their exercise technique. They performed the prescribed exercises with the app’s guidance alone (see Multimedia Appendix 1), without the virtual avatar or sensor-based corrections. All other aspects of the rehabilitation program were kept consistent between the two groups.

### Outcome Measures and Data Collection

The primary outcome was the Hip Disability and Osteoarthritis Outcome Score (HOOS), measured at 24 weeks postsurgery as a comprehensive patient-reported index of hip-related health status [7]. Secondary outcomes were assessed at follow-up visits and included the 5 HOOS subscale scores (pain, symptoms, activities of daily living [ADL], sport/recreation, and hip-related quality of life), objective functional performance tests (the timed up and go [TUG] test and the Berg Balance Scale), generic health-related quality of life measures (36-item Short Form Health Survey [SF-36] physical and mental component summary scores), and psychological status measures (Hospital Anxiety and Depression Scale [HADS] anxiety subscale [HADS-A] and depression subscale [HADS-D]) [8-11]. Patient satisfaction with the rehabilitation program was evaluated at 12 weeks and 24 weeks using a 4-item Likert-scale questionnaire addressing convenience, ease of use and access, perceived helpfulness, and communication with the care provider (each item scored from 1 to 5, with higher scores indicating greater satisfaction).

Cost outcomes were analyzed from a societal perspective capturing direct medical costs (including the cost of the wearable sensor hardware and setup for the IMU group), direct nonmedical costs, and opportunity costs (lost wages and productivity) obtained from hospital billing records and participant self-reports. All costs were converted to 2024 Chinese Yuan (¥), with costs incurred before 2024 adjusted to 2024 price levels using the national consumer price index and any costs occurring beyond 1 year postsurgery discounted at an annual rate of 3%. A currency exchange rate of ¥1=US $0.15 is applicable.

Adherence to the rehabilitation program was measured using multiple indicators collected throughout the 24-week study period. These included the number of prescribed exercise sessions completed, the number of weekly app-based assessments attended, and the number of scheduled weekly follow-up phone calls successfully completed in each group.

Adverse events (AEs) and serious AEs (SAEs) were closely tracked as part of safety monitoring from enrollment through the full 24-week trial. Participants were instructed to report any AEs or SAEs either through the study’s mobile app (which included a “Report a Problem” feature) or via a phone call to the research team. For this study, an AE was defined as any untoward or unintended medical occurrence during the trial, regardless of whether it was considered related to the intervention. An SAE was defined using standard criteria as any adverse event that resulted in death, was life-threatening, required or prolonged hospitalization, caused significant disability or incapacity, or captured any other important medical event that necessitated intervention to prevent such outcomes. Follow-up assessments were conducted at 6 weeks, 12 weeks, and 24 weeks postsurgery in the outpatient clinic. Two trained physiotherapists conducted all outcome measurements at each visit. Participants completed patient-reported outcome measures, including HOOS, HADS, and SF-36, using paper questionnaires. All performance-based outcome tests were administered in person by the physiotherapists during each follow-up visit.

### Sample Size Calculation

The sample size was determined to provide sufficient power to detect a clinically meaningful difference in the primary outcome (HOOS) between groups. Based on published data in THA populations, an improvement of roughly 8 to 10 points on the HOOS represents the minimum clinically important difference in patient-reported hip function after surgery. We therefore set a 10-point difference in HOOS as the target effect size for this trial. To inform the variability estimate, we reviewed prior studies reporting HOOS outcomes and found that the SD of change scores is approximately 15 to 20 points in this context. Using these assumptions and a 2-sided significance level of .05 with 80% statistical power, we calculated that 86 patients per group would be required to detect a 10-point difference in HOOS between the intervention and control arms. This calculation was based on a 2-sample comparison of means for the primary outcome. To account for an anticipated attrition rate of about 25% (loss to follow-up and dropouts), the sample size was inflated accordingly. We aimed to recruit approximately 115 participants per arm to ensure that the study would remain adequately powered for the primary endpoint after accounting for potential dropouts. This sample size was also expected to provide sufficient power for key secondary outcomes while maintaining a feasible recruitment target.

### Randomization, Allocation, and Blinding

An independent statistician generated the allocation sequence using block randomization with randomly varying block sizes (between 4 and 6), achieving a 1:1 allocation ratio to the 2 study groups. Allocation concealment was maintained by placing assignments in sequentially numbered, opaque, sealed envelopes. Randomization was implemented after participants completed baseline assessments. At that time, the enrolling investigator opened the next envelope in sequence to reveal the participant’s group assignment. The allocation list was kept secure by the statistician and was not accessible to investigators or clinicians responsible for recruitment and treatment, ensuring that allocations remained concealed until the point of assignment. Due to the nature of the intervention, participants and treating physiotherapists were not blinded to group allocation; however, all outcome assessors and data analysts remained blinded to group assignments throughout the study to minimize the risk of bias.

### Statistical Analysis

Baseline characteristics, outcome measures, and treatment adherence data were summarized using appropriate descriptive statistics (mean and SD for continuous variables; frequencies and percentages for categorical variables). We applied the Shapiro-Wilk test to formally evaluate normality [12]. A nonsignificant Shapiro-Wilk test (*P*>.05) indicated no evidence against normality. Any major deviations would have prompted use of transformations or nonparametric methods, but no such issues arose in this study. All hypothesis tests were 2-sided with a significance level of 5%. Intention-to-treat (ITT) analyses were primary, including all participants as randomized.

For the primary outcome (HOOS at 24 weeks postrandomization), an adjusted 2-sided analysis was conducted in the ITT population. The primary between-group comparison of HOOS at 24 weeks was based on a linear mixed effects model treating the repeated HOOS measurements as the dependent outcome. This model included fixed effects for treatment group, time, and the treatment-by-time interaction, with baseline HOOS score, age, and sex entered as covariates to improve precision. A random intercept for each participant was incorporated to account for within-subject correlation over time. As this was a single-center trial, no random center effect was needed. The adjusted mean difference in HOOS at 24 weeks between the 2 groups was estimated from this model with a 2-sided 95% CI. The primary hypothesis was evaluated using the *P* value associated with the treatment group effect (difference in outcomes between groups) at the 24-week time point. Model assumptions (such as normality of residuals and homogeneity of variance) were checked, and analyses were robust to these assumptions or used appropriate transformations if necessary.

Continuous secondary outcomes were analyzed with similar linear mixed effects models appropriate for their distribution. Each continuous outcome measured over time was examined for overall group differences and trajectory over the follow-up period. These models included the same fixed effects (group, time, and their interaction) and participant-level random intercepts, adjusting for baseline values when available. From each model, we obtained estimates of the mean outcome in each group at each follow-up time and the mean differences between groups with 95% CIs. Group differences at each time point were derived from interaction terms or post hoc contrasts. For binary categorical outcomes, comparisons between groups were made using *χ*² tests. These dichotomous results were reported as risk differences or relative risks with corresponding 95% CIs. All significance tests were 2-sided, and a *P* value <.05 was considered statistically significant for each individual outcome analysis. To control for inflated Type I error across secondary endpoints, a Bonferroni correction was applied to all secondary outcome comparisons. The primary outcome (HOOS overall at 24 weeks) was tested at a 2-sided significance level of .05 as the single confirmatory analysis. For secondary outcomes, the family-wise alpha was divided by the total number of secondary comparisons (47 tests across 15 secondary outcome measures at up to 3 time points, plus 2 nonprimary time points for HOOS overall), yielding a Bonferroni-adjusted significance threshold of *P*<.001. Both unadjusted and Bonferroni-adjusted significance levels are reported for transparency.

A comprehensive cost-effectiveness analysis was also performed alongside the clinical outcomes. Total health care costs for each participant over the 24-week follow-up were calculated, incorporating intervention costs and any other relevant medical expenditures. The difference in mean total cost between the 2 groups was then evaluated using appropriate between-group comparisons (with nonparametric bootstrap methods applied to account for skewed cost data). We plotted the joint uncertainty in costs and effects on a cost-effectiveness plane to visualize the difference in mean cost against the difference in effectiveness (primary outcome improvement) for the digital rehabilitation versus control. If the digital intervention demonstrated lower average cost while yielding no worse outcome, it was considered a dominant strategy (more cost-effective). In cases where the digital program had higher cost or marginally different outcomes, an incremental cost-effectiveness ratio (ICER) was computed to quantify the additional cost per unit of benefit gained. Uncertainty in the cost-effectiveness results was explored by nonparametric bootstrapping (10,000 resamples), which produced a cloud of incremental cost-effect pairs on the cost-effectiveness plane and allowed construction of cost-effectiveness acceptability curves. These acceptability curves illustrate the probability that the digital program is cost-effective compared with the control across a range of willingness-to-pay thresholds for improvement in outcome.

Missing data were handled using both ITT and per-protocol (PP) analysis strategies. In the ITT analysis, all participants were retained in their originally assigned groups for analysis, regardless of protocol adherence or missing follow-up data. The linear mixed effects model used all available observations from each participant under a missing-at-random assumption, thereby inherently accommodating incomplete follow-up without requiring formal imputation. In contrast, the PP analysis included only those participants who completed all scheduled follow-up assessments, thereby using a complete-case approach. It is important to note that missingness in our dataset was holistic at the follow-up level: If a patient was lost to follow-up at a given time point, all variables for that visit were missing for that patient. There was no item-level missingness within an attended visit. Consequently, missing data occurred only at the participant-visit level, and all available data from each participant’s other time points were still used in the mixed model under the ITT approach. Because entire follow-up visits were missing for dropouts and not selective outcome omissions, we did not perform any statistical imputation for missing values. In addition, given this pattern of whole-visit missingness, a formal test for data missing completely at random (such as the Little MCAR test) was not applicable, as that test is designed for detecting patterns of sporadic item-level missingness across variables rather than the uniform visit-level dropout observed in this trial.

## Results

### Participants

**Figure 1.**
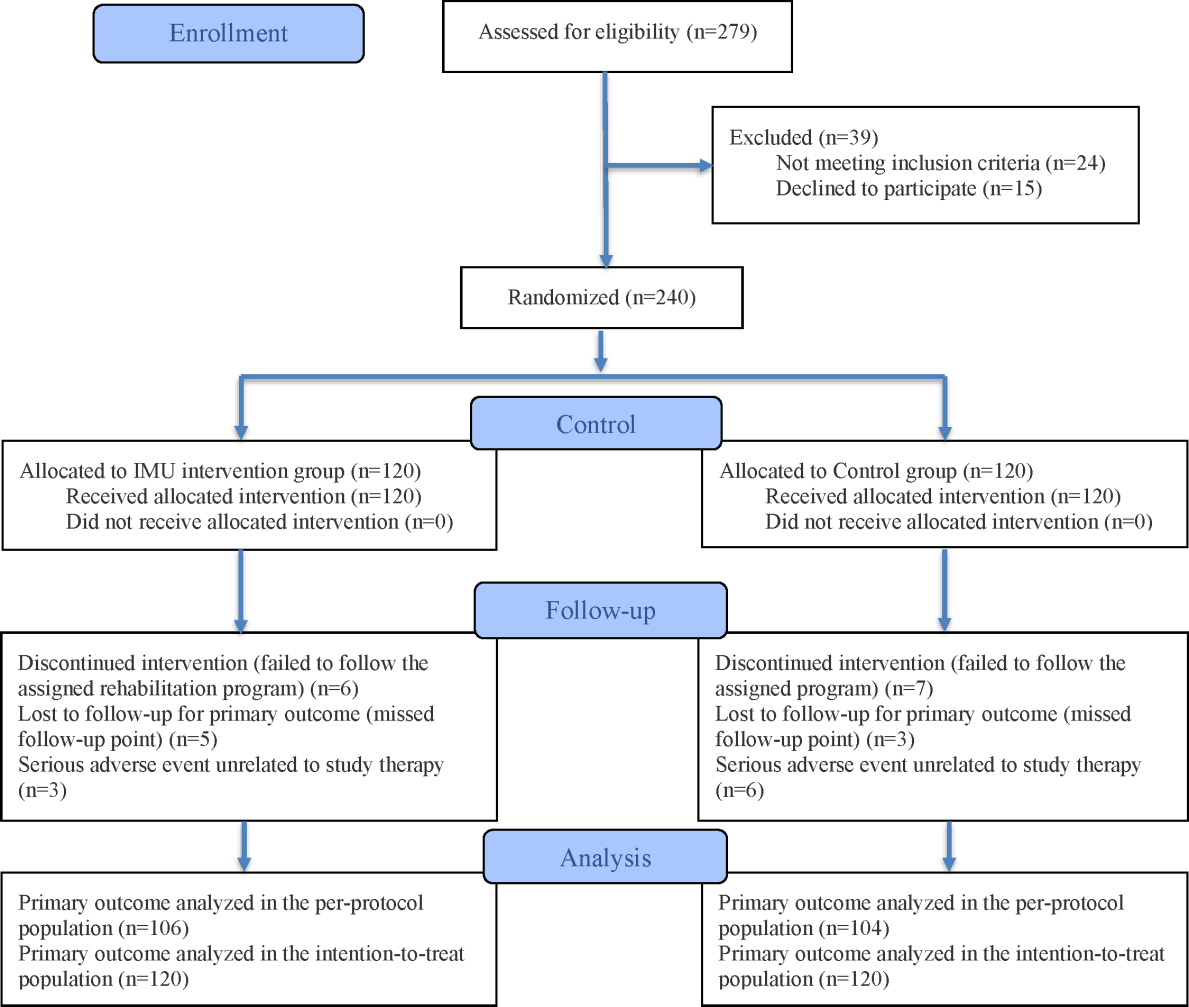
Flow diagram of the study. IMU: inertial measurement unit.

We evaluated 279 patients scheduled for primary THA, and 240 were randomized. The flowchart of our study is shown in Figure 1. The baseline characteristics were comparable between the IMU and control groups. Men accounted for 62 (62/120, 51.7%) participants in the IMU group and 56 (56/120, 46.7%) participants in the control group (*P*=.44); the mean age was 70 years versus 69 years (*P*=.46); mean BMI was 22 kg/m² versus 22 kg/m² (*P*=.31; Table 1). Paracetamol or nonsteroidal anti-inflammatory drugs were taken by 17 (17/120, 14.2%) in the intervention group versus 18 (18/120, 15%) in the control group (*P*=.86). In addition, 62% (74/120, 61.7% vs 72/120, 60%) had the left hip as the surgical side (*P*=.79). There were no significant differences in functional tests nor patient-reported outcome measures.

Sensitivity analysis using the PP population showed similar results as those of the ITT population (Table S1 in Multimedia Appendix 2).

**Table 1.** Baseline characteristics of the inertial measurement unit (IMU) group and control group (intention-to-treat population).

Characteristics	IMU (n=120)	Control group (n=120)	*P* value
Demographics			
Gender (male), n (%)	62 (51.7)	56 (46.7)	.44
Age (years), mean (SD)	70 (5.14)	69 (5.11)	.46
BMI (kg/m^2^), mean (SD)	22 (2.01)	22 (1.86)	.31
Occupation, n (%)			.67
Manual worker	84 (70)	87 (72.5)	
Nonmanual worker	36 (30)	33 (27.5)	
Education level, n (%)			.77
Lower than high school	89 (74.2)	87 (72.5)	
Equal/higher than high school	31 (25.8)	33 (27.5)	
Insurance type, n (%)			.38
Government	85 (70.8)	80 (66.7)	
Commercial	11 (9.2)	18 (15)	
Self-financed	24 (20)	22 (18.3)	
Current smoker, n (%)	42 (35)	48 (40)	.42
Current alcohol use, n (%)	32 (26.7)	29 (24.2)	.66
Paracetamol and NSAID^a^ use, n (%)	17 (14.2)	18 (15)	.86
Side of hip (left), n (%)	74 (61.7)	72 (60)	.79
Living alone (yes), n (%)	76 (63.3)	75 (62.5)	.89
Function, mean (SD)			
HOOS^b^			
Pain	41 (4.30)	40 (5.08)	.26
Symptoms	40 (5.38)	40 (5.31)	.67
Function in ADLs^c^	41 (4.85)	41 (4.40)	.82
Sports and recreation	25 (3.16)	25 (3.52)	≥.99
Hip-related quality of life	25 (3.52)	25 (3.73)	.75
Overall	38 (2.63)	38 (2.43)	.56
TUG^d^ test (seconds)	31 (4.44)	30 (4.80)	.30
Berg Balance Scale	35 (3.51)	35 (3.84)	.41
PROMs^e^			
SF36^f^ physical component summary	25 (4.98)	25 (4.83)	.84
SF36 mental component summary	41 (5.79)	40 (6.44)	.87
HADS^g^ anxiety subscale	10 (2.03)	10 (2.02)	.92
HADS depression subscale	10 (1.85)	10 (2.08)	.95

a NSAID: nonsteroidal anti-inflammatory drug.

b HOOS: Hip Disability and Osteoarthritis Outcome Score.

c ADLs: activities of daily living.

d TUG: timed up and go.

e PROMS: patient-reported outcome measures.

f 36-item Short Form Health Survey.

g HADS: Hospital Anxiety and Depression Scale.

### Primary and Secondary Outcomes

The primary outcome (HOOS) improved significantly more in the IMU group than in the control group at all follow-ups. The estimated between-group difference in the overall HOOS (IMU minus control) was 2.021 (95% CI 1.445‐2.598) points at 6 weeks (*P*<.001), 1.721 (95% CI 0.896‐2.545) points at 12 weeks (*P*<.001), and 1.298 (95% CI 0.313‐2.282) points at 24 weeks (*P*=.01). All 5 HOOS subscales showed a pattern of greater early improvement with the IMU intervention. At 6 weeks, 4 of the 5 subscales (pain, symptoms, sport/recreation, and hip-related quality of life) had significant between-group differences after Bonferroni correction (all *P*<.001), with coefficients ranging from 2.334 to 3.327 points. The HOOS ADL subscale difference at 6 weeks (1.208 points, 95% CI 0.283 to 2.134; *P*=.01) did not survive Bonferroni correction. At 12 weeks, none of the individual HOOS subscale differences remained significant after correction for multiple comparisons (unadjusted *P* values ranged from .002 to .047). By 24 weeks, all subscale differences had further narrowed and were not statistically significant at either the unadjusted or adjusted level (all *P*>.13), except for HOOS pain, which had a nominal difference of 1.501 (95% CI 0.084 to 2.918) points (unadjusted *P*=.04) that did not survive Bonferroni correction. In terms of objective function, the IMU group completed the TUG faster than controls by 1.198 (95% CI 0.789 to 1.607) seconds at 6 weeks (*P*<.001) and 1.131 (95% CI 0.557 to 1.705) seconds at 12 weeks (*P*<.001), both of which remained significant after Bonferroni correction. The 0.901-second (95% CI 0.237 to 1.565) difference at 24 weeks was nominally significant (unadjusted *P*=.008) but did not survive Bonferroni correction. Berg Balance Scale scores were numerically higher in the IMU group at all time points (0.300 to 0.431 points), but none of these differences reached the Bonferroni-adjusted threshold (unadjusted *P* values of .004, .02, and .16 at 6 weeks, 12 weeks, and 24 weeks, respectively). For generic patient-reported outcomes, the SF-36 physical component summary was higher in the IMU group by 3.821 (95% CI 3.315 to 4.326) points at 6 weeks, 2.626 (95% CI 1.913 to 3.338) points at 12 weeks, and 1.949 (95% CI 1.144 to 2.753) points at 24 weeks (all *P*<.001; all surviving Bonferroni correction). The SF-36 mental component summary showed a similar pattern (3.239, 95% CI 2.881 to 3.597; 2.272, 95% CI 1.771 to 2.773; and 1.726, 95% CI 1.117 to 2.334 points at 6 weeks, 12 weeks, and 24 weeks, respectively; all *P*<.001; all surviving Bonferroni correction). Psychological outcomes also consistently favored the IMU arm across all time points and remained significant after Bonferroni correction: The IMU group had lower HADS-A scores by 0.537 (95% CI 0.470 to 0.604), 0.418 (95% CI 0.310 to 0.527), and 0.363 (95% CI 0.220 to 0.506) points at 6 weeks, 12 weeks, and 24 weeks, respectively (all *P*<.001), and lower HADS-D scores by 0.501 (95% CI 0.435 to 0.566), 0.370 (95% CI 0.260 to 0.481), and 0.307 (95% CI 0.163 to 0.450) points at 6 weeks, 12 weeks, and 24 weeks, respectively (all *P*<.001). Patient satisfaction with the rehabilitation program was high in both groups, with no significant between-group differences in any domain at 12 weeks or 24 weeks (all *P*>.23). Detailed results of the primary and secondary outcomes are shown in Tables2  3. Sensitivity analyses in the PP population yielded results consistent with the ITT findings, reinforcing the robustness of the observed treatment effects (Tables S2-S3 in Multimedia Appendix 2).

**Table 2.** Unadjusted changes in outcomes for the inertial measurement unit (IMU) and control groups at the 6-week, 12-week, and 24-week postsurgical follow-ups based on intention-to-treat analysis.

Outcome	6 weeks postsurgery, mean (SD)^a^			12 weeks postsurgery, mean (SD)^a^			24 weeks postsurgery, mean (SD)^a^		
	IMU (n=120)	Control group (n=120)	*P* value^b^	IMU (n=120)	Control group (n=120)	*P* value^b^	IMU (n=120)	Control group (n=120)	*P* value^b^
Functional outcomes^c^									
HOOS^d^									
Pain	13.27 (8.10)	8.58 (4.82)	<.001	23.88 (9.28)	23.46 (9.42)	.74	32.65 (10.20)	32.36 (10.93)	.84
Symptoms	17.54 (8.18)	11.90 (7.56)	<.001	29.21 (10.61)	28.70 (12.92)	.75	37.85 (11.58)	37.98 (12.93)	.94
Function in ADLs^e^	15.85 (8.21)	13.31 (5.75)	.008	28.77 (10.68)	26.75 (9.32)	.14	35.59 (10.78)	35.07 (10.01)	.71
Sports and recreation	22.17 (10.34)	15.28 (9.60)	<.001	41.13 (15.77)	40.77 (11.84)	.85	51.53 (17.64)	51.61 (12.20)	.97
Hip-related quality of life	21.66 (10.96)	17.27 (7.40)	.001	42.43 (16.09)	42.11 (13.39)	.87	54.27 (15.95)	55.00 (14.52)	.72
Overall	16.62 (5.13)	12.57 (2.98)	<.001	30.18 (5.88)	29.13 (5.12)	.16	38.58 (6.06)	38.42 (6.09)	.85
TUG^f^ test (seconds)	−6.19 (3.72)	−3.62 (3.11)	<.001	−8.96 (4.06)	−7.70 (3.77)	.02	−11.39 (4.30)	−10.83 (4.57)	.35
Berg balance test	5.19 (2.58)	4.46 (1.90)	.02	7.61 (2.68)	7.15 (2.23)	.17	10.20 (2.82)	10.31 (2.56)	.76
PROMs^g^									
SF-36^h^									
Physical component summary	30.00 (2.81)	22.50 (2.51)	<.001	42.34 (3.86)	42.32 (3.69)	.96	50.09 (4.77)	50.09 (4.39)	≥.99
Mental component summary	20.07 (2.12)	13.64 (2.12)	<.001	25.23 (4.37)	25.13 (3.47)	.85	33.15 (4.36)	33.16 (3.48)	.98
HADS^i^									
Anxiety subscale (HAD-A)	−2.57 (0.50)	−1.48 (0.50)	<.001	−3.43 (1.51)	−3.29 (0.83)	.39	−4.43 (1.51)	−4.25 (0.91)	.29
Depression subscale (HAD-D)	−2.48 (0.50)	−1.44 (0.50)	<.001	−3.58 (1.28)	−3.50 (1.11)	.64	−4.59 (1.29)	−4.47 (1.11)	.49
Patients’ evaluation of the current intervention									
Convenience	0.00 (0.23)	−0.01 (0.28)	.80	4.75 (1.05)	0.00 (0.27)	<.001	0.00 (0.23)	0.01 (0.29)	.80
Ease of use/access	0.01 (0.21)	−0.01 (0.25)	.57	4.75 (1.05)	0.00 (0.27)	<.001	0.00 (0.27)	−0.01 (0.29)	.81
Helpfulness	0.00 (0.23)	−0.01 (0.25)	.78	4.75 (1.05)	−0.01 (0.25)	<.001	0.00 (0.27)	−0.02 (0.27)	.62
Communication with the care provider	0.00 (0.23)	0.00 (0.27)	≥.99	4.74 (1.05)	0.00 (0.27)	<.001	−0.01 (0.25)	0.01 (0.25)	.60

a The mean change from baseline for each group.

b Between-group differences.

c Functional outcomes included primary endpoints and secondary performance-based tests.

d HOOS: Hip Disability and Osteoarthritis Outcome Score.

e ADLs: activities of daily living.

f TUG: timed up and go.

g PROMs: patient-reported outcome measures.

h 36-Item Short Form Health Survey.

i HADS: Hospital Anxiety and Depression Scale.

**Table 3.** Effectiveness estimates from linear mixed effects models for the inertial measurement unit (IMU) and control groups at the 6-week, 12-week, and 24-week follow-ups based on the intention-to-treat analysis.

Outcome^a^	6 weeks postsurgery		12 weeks postsurgery		24 weeks postsurgery	
	Coefficient (95% CI)^b^	*P* value	Coefficient (95% CI)^b^	*P* value	Coefficient (95% CI)^b^	*P* value
Functional outcomes						
HOOS^c^						
Pain	2.441 (1.632 to 3.250)	<.001	1.874 (0.702 to 3.045)	.002	1.501 (0.084 to 2.918)	.04
Symptoms	2.683 (1.638 to 3.727)	<.001	1.973 (0.415 to 3.531)	.01	1.416 (−2.191 to 3.271)	.14
Function in ADLs^d^	1.208 (0.283 to 2.134)	.01	1.390 (0.016 to 2.765)	.047	1.079 (−0.566 to 2.724)	.20
Sports and recreation	3.327 (1.991 to 4.664)	<.001	2.276 (0.426 to 4.126)	.02	1.555 (−0.652 to 3.762)	.17
Hip-related quality of life	2.334 (1.152 to 3.517)	<.001	1.873 (0.035 to 3.711)	.046	1.321 (−0.864 to 3.505)	.24
Overall	2.021 (1.445 to 2.598)	<.001	1.721 (0.896 to 2.545)	<.001	1.298 (0.313 to 2.282)	.01
TUG^e^ test (seconds)^f^	−1.198 (−1.607 to −0.789)	<.001	−1.131 (−1.705 to −0.557)	<.001	−0.901 (−1.565 to −0.237)	.008
Berg Balance Scale^f^	0.393 (0.125 to 0.661)	.004	0.431 (0.065 to 0.797)	.02	0.300 (−0.118 to 0.718)	.16
PROMs^g^						
SF-36^h^^,f^						
Physical component summary	3.821 (3.315 to 4.326)	<.001	2.626 (1.913 to 3.338)	<.001	1.949 (1.144 to 2.753)	<.001
Mental component summary	3.239 (2.881 to 3.597)	<.001	2.272 (1.771 to 2.773)	<.001	1.726 (1.117 to 2.334)	<.001
HADS^i^^,^^f^						
Anxiety subscale (HAD-A)	−0.537 (−0.604 to −0.470)	<.001	−0.418 (−0.527 to −0.310)	<.001	−0.363 (−0.506 to −0.220)	<.001
Depression subscale (HAD-D)	−0.501 (−0.566 to −0.435)	<.001	−0.370 (−0.481 to −0.260)	<.001	−0.307 (−0.450 to −0.163)	<.001
Patients’ evaluation of the current intervention^f^						
Convenience	0.006 (−0.018 to 0.030)	.65	0.007 (−0.015 to 0.029)	.54	0.005 (−0.014 to 0.025)	.59
Ease of use/access	0.008 (−0.013 to 0.029)	.44	0.012 (−0.008 to 0.032)	.23	0.011 (−0.008 to 0.031)	.24
Helpfulness	−0.003 (−0.026 to 0.021)	.82	0.000 (−0.020 to 0.021)	.97	0.000 (−0.021 to 0.022)	.97
Communication with the care provider	0.005 (−0.015 to 0.026)	.61	0.002 (−0.018 to 0.021)	.88	−0.001 (−0.020 to 0.018)	.93

a All outcome measures were adjusted for baseline values in the model.

b Estimated between-group difference in the change from baseline (IMU group minus control group) for the specified outcome, with positive coefficients indicating higher scores in the IMU group compared with the control group and negative values indicating lower scores in the IMU group.

c HOOS: Hip Disability and Osteoarthritis Outcome Score.

d ADLs: activities of daily living.

e TUG: timed up and go.

f Secondary outcomes with *P*<.001 remained significant after Bonferroni correction. A Bonferroni correction was applied to 47 secondary comparisons, yielding an adjusted significance threshold of *P*<.001.

g PROMs: patient-reported outcome measures.

h 36-item Short Form Health Survey.

i HADS: Hospital Anxiety and Depression Scale.

### Cost Measurements

The mean 24-week total cost per patient was ¥87,967.32 (SD ¥8854.19) in the IMU group versus ¥94,396.30 (SD ¥10,037.74) in the control group (*P*=.32). The cost composition differed between groups: IMU participants had significantly lower in-hospital costs (¥69,534.95, SD ¥5394.69 vs ¥70,359.45, SD ¥5043.84; *P*<.001) and primary care costs (¥250.00, SD ¥38.14 vs ¥1503.18, SD ¥186.13; *P*<.001). Nonmedical expenses were lower with IMU, including paid home care (¥1178.73, SD ¥362.12 vs ¥1451.95, SD ¥357.74; *P*<.001), transportation (¥800.06, SD ¥138.89 vs ¥1968.67, SD ¥273.82; *P*<.001), and nutrition (¥4505.32, SD ¥479.00 vs ¥5093.08, SD ¥519.96; *P*<.001). Patient productivity losses were also lower (¥1599.77, SD ¥6994.72 vs ¥1829.44, SD ¥6618.91; *P*<.001). The detailed results are shown in Table 4.

The IMU intervention’s incremental cost was –¥6428.98 (IMU less costly). Correspondingly, most ICERs were negative, though the overall cost difference between groups was not statistically significant (¥87,967.32 vs ¥94,396.30, *P*=.32). For the primary outcome (HOOS overall), the incremental effect was +1.298 (95% CI 0.313 to 2.282) points, with an ICER of –¥4954 per point. TUG time decreased by 0.901 (95% CI 0.237 to 1.565) seconds (ICER ¥7136.79 per second), and the Berg Balance Scale increased by 0.300 (95% CI –0.118 to 0.718) points (ICER –¥21,451.43 per point). SF-36 physical component summary and mental component summary improved by 1.949 (95% CI 1.144 to 2.753) points (ICER –¥3299.31 per point) and 1.726 (95% CI 1.117 to 2.334) points (ICER –¥3725.41 per point), respectively. The detailed results, including for the HOOS subscales, are shown in Table 5. PP sensitivity analyses (Tables S4 and S5 in Multimedia Appendix 2) yielded similar cost-effectiveness results.

**Table 4.** Average total cost per patient in the inertial measurement unit (IMU) group and control group during the 24 weeks after the surgery.

Cost category^a^ (¥^b^)	IMU (n=120)	Control group (n=120)	*P* value
Medical costs^c^			
Costs while in the hospital (including the hospital stay, surgery fee, medication, implants during surgery)	69,534.95 (5394.69)	70,359.45 (5043.84)	<.001
Physical therapist costs (including the instrument)	6980.00 (0.00)	8447.88 (1046.07)	.24
Primary care costs	250.00 (38.14)	1503.18 (186.13)	<.001
Nonmedical costs^d^			
Paid home costs	1178.73 (362.12)	1451.95 (357.74)	<.001
Transportation costs	800.06 (138.89)	1968.67 (273.82)	<.001
Nutrition costs	4505.32 (479.00)	5093.08 (519.96)	<.001
Opportunity costs^e^			
Lost wages for patients	1599.77 (6994.72)	1829.44 (6618.91)	<.001
Lost wages for families	3118.49 (4461.09)	3742.65 (4743.85)	.80
Total cost	87,967.32 (8854.19)	94,396.30 (10,037.74)	.32

a All relevant costs were captured from a societal perspective and were obtained from hospital billing records and patient self-reports where needed (wage losses). All costs are reported in 2024 Chinese Yuan, as 2024 was the end of the enrollment period; costs incurred in earlier years were adjusted to 2024 price levels using the consumer price index. Costs and outcomes occurring beyond the initial year were discounted at an annual rate of 3% to reflect time preference, consistent with standard practice in health economic evaluations.

b A currency exchange rate of ¥1=US $0.15 is applicable.

c Hospital stay; primary and secondary care; rehabilitation and physical therapy costs, including wearable sensor hardware and setup for the IMU group; and medication costs.

d Direct costs related to care (transportation for medical visits and any specialized nutritional support during recovery).

e Indirect costs due to productivity loss (lost wages for patients during recovery or disability, and lost income for family members/caregivers, if applicable).

**Table 5.** Incremental cost-effectiveness ratio (ICER), defined as the incremental cost divided by the incremental effect, representing the additional cost per unit improvement in outcome for the inertial measurement unit (IMU) group versus the control group.

Items	Main analysis: mixed effects (95% CI)
Incremental cost (¥)	−6428.98
Incremental HOOS^a^ pain	1.501 (0.084 to 2.918)
Incremental HOOS symptoms	1.416 (−2.191 to 3.271)
Incremental HOOS function in ADL^b^	1.079 (−0.566 to 2.724)
Incremental HOOS sports and recreation	1.555 (−0.652 to 3.762)
Incremental HOOS hip-related quality of life	1.321 (−0.864 to 3.505)
Incremental HOOS overall	1.298 (0.313 to 2.282)
Incremental TUG^c^ test (seconds)	−0.901 (−1.565 to −0.237)
Incremental Berg Balance Scale	0.300 (−0.118 to 0.718)
Incremental SF36^d^-physical component summary	1.949 (1.144 to 2.753)
Incremental SF36-mental component summary	1.726 (1.117 to 2.334)
Incremental HADS^e^ anxiety subscale (HAD-A)	−0.363 (−0.506 to −0.220)
Incremental HADS depression subscale (HAD-D)	−0.307 (−0.450 to −0.163)
Incremental convenience	0.005 (−0.014 to 0.025)
Incremental ease of use/access	0.011 (−0.008 to 0.031)
Incremental helpfulness	0.000 (−0.021 to 0.022)
Incremental communication with the care provider	−0.001 (−0.020 to 0.018)
ICER^f^ (¥)	
ICER HOOS pain	−4282.69
ICER HOOS symptoms	−4540.12
ICER HOOS function in ADL	−5958.28
ICER HOOS sports and recreation	−4134.15
ICER HOOS hip-related quality of life	−4868.05
ICER HOOS overall	−4954.13
ICER TUG test (seconds)	7136.79
ICER Berg balance test	−21,451.43
ICER SF36-physical component summary	−3299.31
ICER SF36-mental component summary	−3725.41
ICER HADS anxiety subscale (HAD-A)	17,704.97
ICER HADS depression subscale (HAD-D)	20,954.26
ICER convenience	−1,204,447.61
ICER ease of use/access	−559,377.37
ICER helpfulness	−14,782,662.68
ICER communication with the care provider	7,845,003.05

a HOOS: Hip Disability and Osteoarthritis Outcome Score.

b ADLs: activities of daily living.

c TUG: Timed Up and Go test.

d 36-item Short Form Health Survey.

e HADS: Hospital Anxiety and Depression Scale.

f Incremental effects for primary outcome measures for the 24-week follow-up under both the intention-to-treat and per-protocol analyses. A negative ICER suggests that the IMU intervention achieved numerically better outcomes at a numerically lower cost compared with the control group; however, the statistical significance of both the cost and effect differences should be considered when interpreting these ratios.

### Adherence and AEs

Adherence to the intervention was high in both groups, with the IMU group completing more of the 60 prescribed exercise sessions (mean 51.6, SD 9.3 vs mean 48.7, SD 10.6; *P*=.002), attending more of the 12 weekly app-based assessments (mean 10.5, SD 1.5 vs mean 9.5, SD 1.8; *P*<.001), and completing more of the 12 follow-up phone calls (mean 10.8, SD 1.6 vs mean 9.3, SD 1.8; *P*<.001) than the control group (Table S6 in Multimedia Appendix 2). AEs were infrequent, occurring in 7 patients (7/120, 5.8%) in the IMU group and 9 patients (9/120, 7.5%) in the control group; most AEs were minor and considered related to treatment (Table S7 in Multimedia Appendix 2).

## Discussion

This RCT demonstrated that integrating a wearable sensor into a home-based digital rehabilitation program after THA resulted in significantly greater improvements in patient outcomes compared with a standard digital program without sensor feedback. Specifically, the sensor group achieved significantly larger gains in the primary hip outcome measure and multiple secondary functional and quality-of-life outcomes, particularly during early recovery, although most between-group differences narrowed by the final 24-week follow-up. These statistical differences were attained without increasing health care costs. Total 24-week costs were comparable between groups. Although several individual cost components were numerically lower in the sensor arm, the overall cost difference did not reach statistical significance, suggesting that the sensor-based program was cost-neutral rather than definitively cost-saving. Adherence to the prescribed rehabilitation was high in both groups, with the sensor group completing a higher proportion of exercise sessions and assessments than the control group.

Our findings suggest that augmenting home-based digital rehabilitation with real-time sensor feedback can confer modest gains in early postoperative recovery, though the clinical significance of these gains warrants careful interpretation. The IMU‐enhanced training led to faster improvements in hip function and mobility during the initial weeks, although not all secondary hip-specific outcomes survived Bonferroni correction for multiple comparisons, which is consistent with other orthopedic populations. For example, a recent RCT with patients with diabetes who had undergone total knee arthroplasty demonstrated that adding IMU sensor feedback to telerehabilitation significantly improved functional outcomes compared with telerehabilitation alone [13]. More broadly, our results align with a growing body of evidence that technology-assisted or remote rehabilitation is at least as effective as standard physiotherapy. Meta-analyses in knee and hip arthroplasty have shown that telerehabilitation can yield equal or even superior short-term improvements in pain, range of motion, strength, and functional scores when compared with traditional in-person programs [14-16]. These early advantages are likely attributable to enhanced exercise technique, engagement, and adherence driven by the biofeedback component. Notably, the between-group differences in hip-specific outcomes narrowed by the 24-week follow-up. After Bonferroni correction, no HOOS subscale nor TUG difference remained significant at 24 weeks. However, the SF-36 and HADS differences persisted through the final follow-up even after correction, suggesting that the sensor-mediated benefits on broader quality of life and psychological well-being were more durable than the hip-specific functional gains. This trajectory mirrors prior reports of musculoskeletal rehabilitation, indicating that initial benefits of more intensive or innovative interventions may diminish over time as all patients progress through natural recovery [15]. Indeed, telerehabilitation appears to facilitate a comparable 6-month outcome to that of conventional care in many settings, supporting its use as a viable alternative without long-term trade-offs [15,16]. It is important to acknowledge that, although the improvements with sensor guidance were statistically significant, their absolute magnitude was relatively small. For instance, the HOOS overall score in the IMU group was only 2 points higher on average, a difference below the typical minimal clinically important difference reported for hip outcomes [12]. This suggests that, although sensor-based digital training accelerates early rehabilitation and subjective recovery, the clinical significance of the advantage is modest. Nevertheless, even slight early gains in function and pain relief can be valuable; achieving milestones sooner may improve patient confidence, expedite return to daily activities, and potentially reduce short-term complication risks. In summary, the interpretation of our findings is consistent with existing evidence: Wearable sensor integration enhances the quality of home exercise performance and adherence, thereby providing a small boost to early postoperative outcomes, while overall recovery at the mid-term remains comparable to traditional rehabilitation approaches [13-16].

From a clinical perspective, these findings support integrating wearable sensor–enhanced telerehabilitation into standard post-THA care. Patients in the sensor-based program achieved faster early gains in functional mobility and quality of life without compromising longer-term outcomes. This accelerated recovery can improve the early postoperative experience and potentially reduce short-term disability. Importantly, the sensor feedback substantially boosted patient adherence to prescribed exercise, a critical determinant of successful rehabilitation, echoing prior reports of higher compliance with telerehab interventions [17]. The approach was also safe and well-received, with similar patient satisfaction and no increase in adverse events compared with usual care. Furthermore, our trial’s economic analysis indicates that delivering home-based digital rehabilitation with wearable sensors is feasible without added cost burden. Although the point estimate suggested numerically lower costs in the sensor group, the overall cost difference was not statistically significant, and shifting rehabilitation into the home may help reduce indirect costs (travel, time off work) and alleviate demands on in-person physiotherapy services [6,17]. With THA volumes rising globally, adopting such technology-assisted home programs could expand rehabilitation capacity, improve access for patients in remote or resource-limited settings, and enhance the value of care by providing efficient, patient-centered recovery support [17].

This study has several notable strengths, including its robust randomized controlled design (with blinded outcome assessments) and comprehensive outcome evaluation encompassing both clinical and economic measures, which bolsters confidence in the findings. However, there are also important limitations. Methodologically, some experimental details lacked clarity or could have been more appropriately standardized, potentially affecting reproducibility. Analytically, the data analysis methods, although rigorous, involved statistical assumptions that may limit their appropriateness or introduce bias or uncertainty. Additionally, the results should be interpreted with caution, as conclusions remain contingent on the study’s context and design. We also strived for scientific accuracy and consistent terminology, but minor ambiguities in usage and definitions may persist. These considerations highlight areas for improvement; future studies will address these concerns by refining methodology and analysis, clarifying terminology, and further validating our findings.

In conclusion, this RCT showed that adding wearable sensor feedback to a home-based digital THA rehabilitation program led to improved early functional outcomes, sustained benefits in patient-reported quality of life and psychological well-being, and significantly higher patient adherence compared with a similar program without sensors while incurring no additional costs. Several early hip-specific functional differences did not survive Bonferroni correction for multiple comparisons, and most HOOS subscale differences had converged by 24 weeks. Although the between-group differences in HOOS were statistically significant, their magnitude remained below the established minimal clinically important difference, tempering the clinical significance of these gains. Nevertheless, the sensor-enhanced approach provided consistent early benefits across multiple outcome domains and substantially improved exercise adherence. The intervention was cost-neutral, suggesting that integrating wearable technology in post-THA rehabilitation can enhance patient engagement and accelerate early recovery without adding to the economic burden.

## Supplementary material

10.2196/93050 Multimedia Appendix 1 Intervention details, including the adaptive training protocols and ongoing adjustments.

10.2196/93050 Multimedia Appendix 2 Characteristics and changes in outcomes in the per-protocol population and patient adherence and adherence in the intention-to-treat population.

10.2196/93050 Checklist 1 CONSORT-EHEALTH (Consolidated Standards of Reporting Trials of Electronic and Mobile Health Applications and Online Telehealth) checklist.

## Abbreviations

ADL: activities of daily living
AE: adverse event
CONSORT: Consolidated Standards of Reporting Trials
HADS: Hospital Anxiety and Depression Scale
HADS-A: Hospital Anxiety and Depression Scale anxiety subscale
HADS-D: Hospital Anxiety and Depression Scale depression subscale
HOOS: Hip Disability and Osteoarthritis Outcome Score
ICER: incremental cost-effectiveness ratio
IMU: inertial measurement unit
ITT: intention-to-treat
PP: per-protocol
RCT: randomized controlled trial
SAE: serious adverse event
SF-36: 36-item Short Form Health Survey
THA: total hip arthroplasty
TUG: timed up and go
