# Supplementary material for: The Efficiency and Cost-Effectiveness of Wearable Sensors in a Digital Physiotherapeutic Total Hip Arthroplasty–Specific Training System for Patients After Total Hip Arthroplasty: Randomized Controlled Trial
Source: JMIR Mhealth Uhealth. 2026 Jul 21;14:e93050. doi: 10.2196/93050 (PMC13387417; doi:10.2196/93050)
Supplement: Multimedia Appendix 1 [file mhealth-v14-e93050-s001.docx]

**Intervention**

**The 12-week standardized post-THA training protocol**

| **Week** | **Assessment** | **Goals** | **Exercises & Frequency** | **Modalities** | **Remarks** |
| --- | --- | --- | --- | --- | --- |
| **Week 1**(Post-op 2–3 weeks) | Initial Evaluation: In-person baseline assessment by physiotherapist (pain levels, incision check, hip ROM, gait with walker). App Check-in: Patient logs pain (0–10) and exercise completion via app. | Early Recovery: Protect healing hip, manage pain/swelling, prevent complications (DVT, dislocation). Begin gentle motion and muscle activation. Achieve independent bed mobility and transfers with assistive device. | Therapeutic Exercises: 5 days/week home program. Emphasis on circulation and activation: ankle pumps, deep breathing, isometric quad and glute sets, gentle heel slides, and hip abduction in supine (within precautions). Short, frequent walks with walker (indoors, e.g. 5 minutes, 2–3x/day). Frequency: Daily sessions (30 minutes) focusing on basics. | Cryotherapy: Ice pack to hip area 15 min after exercises, 2–3× daily to reduce pain and swelling. Compression: Wear compression stockings per surgical protocol for DVT prophylaxis. | Precautions: Maintain surgeon’s hip precautions (e.g. no hip flexion beyond 90° if posterior approach). Use walker for all walking (weight-bearing as tolerated unless otherwise directed). Monitor incision for signs of infection. Emphasize patient education on not pivoting on surgical leg and avoiding low chairs. |
| **Week 2** | Weekly App Assessment: Patient reports pain and difficulty after each session via the app; adherence monitored by therapist remotely. No in-person visit this week unless issues arise. | Mobility & Self-Care: Improve safe ambulation distance with walker (e.g. from 10 to 50+ meters as tolerated). Begin restoring hip ROM (gentle active-assisted range). Increase patient confidence in basic self-care activities (dressing, bathroom) using adaptive equipment. | Exercises: Continue daily home exercise routine (5×/week). Progress exercises slightly: add active-assisted hip flexion and extension (within safe range), gentle standing weight shifts holding a countertop, mini knee bends while holding walker (to engage hip and thigh muscles lightly). Maintain isometrics and add supine straight leg raises if able (with brace or assistance if needed for form). Gait: Increase walking duration to 5-10 minutes 2×/day, using walker, focusing on proper heel-toe pattern. | Thermal Modalities: Warm pack on low back or thigh muscles for 10 minutes before exercise if stiffness is an issue (to promote flexibility). Continue ice to hip after exercise or if swelling occurs. | Remarks: Patient should avoid overexertion, if pain >4/10 or increased swelling after exercise, they should scale back intensity. Emphasize proper walker use (keep upright posture, avoid hopping). Ensure compliance with blood-thinner medications and home safety (remove tripping hazards). No driving yet. Encourage high-protein diet for healing. |
| **Week 3** | Remote Monitoring: Therapist reviews app data (exercise completion rate, sensor feedback if available). Weekly patient-reported outcome (e.g. HOOS Jr short survey via app) to track subjective progress. Phone call check-in if any red flags (high pain or low adherence). | Strength & Gait Initiation: Further improve hip muscle recruitment and begin transitioning towards greater weight-bearing. Goal to wean from walker to a cane if safe by end of week (based on balance and pain). Increase hip ROM gradually (target: 0–90° flexion comfortably if posterior approach). Enhance confidence with basic gait. | Exercises: 5×/week sessions at home, advancing difficulty slightly. Add standing exercises with support: e.g. standing hip abduction (holding onto counter), standing hip extension (leg back) to strengthen glutes. Initiate gentle mini-squats (no more than 45° knee bend) with hands on a stable surface for support, focusing on equal weight on both legs. Increase walking: aim for 10-15 min total walking per day (split into shorter walks). Begin practicing stair navigation (if applicable at home) with rail support, “up with the good, down with the bad” technique. | Modalities as Needed: Continue ice after exercise if swelling or pain flare occurs. Many patients will start reducing ice use by end of week 3 as acute inflammation subsides, but it remains useful after longer walks. If quadriceps activation is poor, clinic may introduce NMES (neuromuscular electrical stimulation) to the thigh briefly during PT sessions to facilitate muscle contraction (optional). | Remarks: If patient demonstrates good balance and leg control, they may progress from walker to a cane late in Week 3 (with therapist or family supervision). Ensure proper cane usage on opposite side of operated hip. Continue to reinforce hip precautions (they still apply). Home environment should be prepared for increasing activity (e.g. practicing walk in yard or hallway). Encourage patient to listen to their body, slight muscle soreness is normal, sharp pain is not. |
| **Week 4** | Monthly In-Person Assessment: Patient comes to clinic for a 4-week evaluation. Measurements: hip ROM (target 100° flexion if no precautions limit it), hip abduction strength (manual muscle test), gait quality and assistive device use, swelling around hip. Outcome measures: Timed Up and Go (TUG) test for basic mobility, 30-second chair stand test if able. Review of app logs and address any exercise form issues. | Milestone Check: Evaluate progress and adjust program. Goals by end of Week 4: independence in household ambulation with a cane if appropriate, ability to stand from chair with minimal use of arms, improved hip muscle strength (grade 3+ out of 5). Pain at rest should be mild (<3/10). Set new goals for next month (e.g. begin outdoor walks, start driving if cleared). | Exercises: Update home exercise plan based on findings. Continue core set of exercises 5×/week, with increased intensity: introduce resistance bands for some movements (e.g. clamshells or standing abduction with band resistance). Add supine bridge exercises (lying on back, bend knees and lift hips) to strengthen glutes and core, if patient can do without pain. Progress squats a bit deeper (as tolerated, maybe to 60° knee bend) and add step-ups: begin practicing stepping up on a single step (4–6 inch height), leading with operated leg, holding a support for balance. Increase balance work: try standing on operated leg for a few seconds with support (building proprioception). Endurance: encourage 15-20 minutes of continuous walking daily by end of week (can be split into 2×10 min). If available, begin stationary cycling with no resistance for 5-10 minutes, to gently improve hip motion and endurance (ensure seat is high to avoid excessive hip flexion). | Modalities: Heat & Ice: If stiff before exercise, apply moist heat pack to hip/thigh 10 min prior to warm up. Continue icing after longer exercise bouts if swelling occurs. Aquatic Therapy (Optional): If patient has access to a pool and incision is fully healed, they can start pool walking or gentle aquatic exercises this week, water provides low-impact resistance and can reduce pain during movement. | Remarks: Discuss with patient and surgeon about clearance for driving (often around 4-6 weeks post-op if off narcotic pain meds and left hip surgery, or right hip if car is automatic and patient feels in control). Emphasize continued safety: use cane until gait is without a significant limp. At this stage, some hip precautions may be eased per surgeon (e.g. many allow bending past 90° after 6 weeks, but continue to avoid extremes until 12 weeks). Patient motivation is key, highlight achievements so far and set expectations for next phase (e.g. longer walks, using a single cane or no device in home by next month). |
| **Week 5** | Weekly App Review: Automated check-ins via app on pain (expected to be decreasing) and exercise difficulty. Therapist remotely monitors that patient is meeting exercise frequency. No new in-person visit this week unless issues. | Building Strength: Focus on improving muscle strength and balance. Aim for more normalized gait pattern (shorten use of cane indoors if possible). Increase activity tolerance (e.g. able to walk 1/4 mile continuously by end of week 6 target). Pain mostly activity-related and manageable with minimal medication. | Exercises: Continue home program 5×/week, progressing reps and resistance. By now, patient should increase repetitions (aim 2–3 sets of 10–15 reps for each exercise if tolerable). Add new exercises for advanced strength: lateral band walks (side-stepping with resistance band at knees), wall squats (back against wall, slide down to slight squat) if appropriate. Balance drills: tandem standing (heel-to-toe stance) or weight shifting with less support to challenge balance (ensure a stable object nearby). Core strengthening: introduce planks or side-bridges modified (if patient is able) to support overall stability. Aerobic: encourage longer walks, possibly start gentle outdoor cycling on flat ground if patient is skilled (with surgeon approval). Frequency: maintain 5 days exercise, plus daily walking for endurance. | Modalities: Ice usage likely much reduced; patient uses it only if swelling after higher activity. If persistent muscle soreness, can alternate heat before exercise and ice after. If patient reports any sharp pain in groin or thigh, advise rest and icing and consider evaluation to rule out issues. Typically no formal modalities needed this week beyond self-management. | Remarks: By this point, many patients feel a significant improvement in daily function. Continue to ensure gait quality, therapist may have instructed patient in cane weaning: try short periods walking without cane on flat, supervised environment to test gait (if no limp and no pain, can gradually discontinue cane by end of next week). Remind not to limp; if they limp without cane, they should continue using it. Reinforce proper exercise form (app videos and feedback help here). Encourage participation in light activities they enjoy (with caution) to keep motivation, e.g. light household chores, guided by pain tolerance. |
| **Week 6** | Midpoint Virtual Check-in: A video call with physiotherapist is scheduled around week 6. Therapist observes patient performing a few key exercises (squat, leg raises, step-up) to ensure proper form and adapts technique if needed. Gait assessment via video: patient walks to check for any lingering deviations. Outcome Measures: Possibly repeat a Timed Up and Go test at home if feasible (therapist times via video) to compare to week 4. Patient completes a HOOS Jr or other survey via app to gauge functional improvement. | Midpoint Targets: At roughly half-way, patient should have near full ROM or at least functional ROM for daily activities (e.g. 0-110° flexion, 20° abduction). Strength of operative leg 60–70% of the non-operative side in key muscle groups. Gait should be markedly improved, goal to walk without cane on level surfaces if not already, and to begin more dynamic tasks. Plan next phase for return to more demanding activities (driving, light sport). | Exercises: Home routine now enters a more advanced phase. Continue prior exercises with further progression: increase resistance band tension or add light ankle weights for leg raises. Introduce closed-chain strengthening: e.g. light leg press if equipment available (or functional equivalent like sit-to-stand from a lower chair height to increase challenge). Step Training: progress from a single step to a stair: practice stair ascent/descent reciprocally if safe (holding railing). Balance/proprioception: single-leg stands for 10+ seconds (with support nearby), begin gentle dynamic balance like shifting weight in a staggered stance or using a balance board (with supervision). Endurance: encourage longer outdoor walks (20+ minutes continuous if possible by end of week 6), perhaps goal of 1/2 mile by now if there are no complications. May start using a treadmill at slow speed if patient prefers indoor (no incline yet). Frequency remains 5× week formal exercise, daily low-impact aerobic activity. | Modalities: Little reliance on passive modalities now. Aquatic Therapy: If patient enjoys pool exercise, they can now do more challenging pool workouts (e.g. leg kicks, pool walking laps) since strength improved, water exercises can supplement land therapy. If any specific issue (like bursitis or tendonitis from increased activity), modalities like ultrasound or TENS could be utilized in clinic for pain relief, but only if needed. | Remarks: Many surgeons lift most movement precautions at 6 weeks if healing is on track; patient may be allowed to tie their shoes, bend forward more normally, confirm with surgeon and ensure patient still uses common sense (avoid extreme positions initially). If not already driving, most can resume driving now (especially if left hip or automatic car and off strong pain meds). Emphasize return to normalizing daily life: can likely do light household activities, shopping with a cart, etc., as long as pain-free. Advise them to still avoid high-impact or high-torque motions. The focus is now on getting stronger and more confident, celebrate progress (e.g. improved TUG time, longer walks) to keep motivation high for the second half of rehab. |
| **Week 7** | Weekly Progress Review: Therapist checks app data and notes if patient is consistently reaching exercise targets. Automated message prompts patient to report any unusual pain or swelling; if reported, therapist contacts patient for guidance. No routine in-person visit this week. | Advanced Function: Emphasize advanced strength and balance. Aim to walk without assistive device even outdoors on flat ground. Improve ability to do functional tasks like getting in/out of a car smoothly, climbing a standard flight of stairs foot-over-foot. Continue to increase hip muscle endurance (e.g. able to do 15+ reps of key exercises). | Exercises: Further progression in the home program, 4–5×/week of focused exercise plus daily general activity. Strength: Increase load: e.g. if using ankle weights (1–2 kg), or heavier resistance bands for hip abduction and extension. Add lunges (forward or static split squats) if balance allows, to build hip and thigh strength in a functional pattern (ensure knee aligned, do shallow lunges first). Step-up height can be increased (from a single stair to a higher step or step stool, focusing on controlled descent to build eccentric strength). Balance: challenge with eyes closed balance stand or gentle side-to-side swaying on a foam surface. Possibly incorporate a simple agility drill like sidestepping or gentle figure-8 walking around obstacles to simulate real-life movements. Endurance: encourage non-stop walking 25–30 minutes. If patient is inclined, they may try cycling outdoors or using an elliptical trainer with low resistance (if they have sufficient hip extension and no pain on it). | Modalities: Generally none required except the occasional ice after an intense exercise day if slight swelling. Patient should be largely self-sufficient in managing soreness (maybe using over-the-counter NSAIDs or ice as needed). In clinic, biofeedback devices might be used if available to fine-tune gait or exercise form (e.g. laser pointer cane to encourage symmetrical steps), though not a traditional modality, it’s an adjunct tool for training. | Remarks: Patient is moving toward higher level activities. Discuss goals like returning to specific hobbies: for example, if the patient golfs, can plan to resume chipping and putting practice by Week 8–10; if they like gardening, can likely do light gardening with raised beds now, etc. Emphasize continuing home exercise compliance even as they feel better, this is when patients might start skipping exercise because daily life feels normalizing, but continued rehab is crucial for optimal outcome. Also remind them to practice proper body mechanics (avoid lifting heavy objects from low positions, etc., until fully rehabilitated). |
| **Week 8** | Second Monthly Assessment (8-week check): In-person visit with PT or surgeon’s team. Evaluate hip ROM (should be near or at full functional range now), muscle strength (hip abductors, extensors should be significantly improved; ideally ≥4/5 manual strength). Perform gait analysis, by Week 8 patient should walk without a limp at moderate pace. Functional tests: e.g. 6-minute walk test to measure endurance gains since baseline, stair climb test if appropriate. Patient completes outcome questionnaires (HOOS Jr, SF-36 or others) for interim progress. Discuss any barriers or pain points. | Refined Goals: By two months, patient should be largely independent in daily living without accommodations. Goals now include return to moderate activities: for instance, comfortable community ambulation (walk several blocks), able to carry groceries, possibly return to light work duties if applicable. Plan remaining rehab to address any deficits (e.g. if balance is still not 100%, put extra focus there). Set goal for 12 weeks: near full strength and confidence in hip, ready for unrestricted activities per surgeon clearance. | Exercises: Home program continues 3–4× per week focusing on any identified weaknesses from assessment. If strength is nearly normal, shift emphasis to more functional training and higher-level balance: e.g. practice walking on uneven surfaces (grass, gravel) to build confidence in hip stability. If patient is interested in sports like golf or biking, introduce sport-specific drills: gentle golf swing motion without a club or on driving range with half-swing; longer outdoor cycling rides as tolerated. Possibly introduce light jogging in a straight line on a soft surface ONLY if patient is young, active, surgeon-approved (most cases jogging is not until 12+ weeks, so usually hold off, walking briskly is fine). Continue progressive resistance: one-legged standing from sitting (pistol squat assisted) if capable, or single-leg leg press in gym. Increase plyometric prep: maybe start mini quick steps in place or side stepping faster to prepare for any future higher impact (only if appropriate for patient’s goals). Endurance: aim that patient can walk 1 mile by end of this week or next, at comfortable pace. Frequency: active most days, formal strength exercises at least every other day. | Modalities: Hardly any traditional modalities indicated at this stage aside from recovery techniques: encourage ice or elevation only if swelling after strenuous new activity. Possibly use compression (sleeve or stocking) during long walks if mild swelling occurs in ankle by day’s end, some patients still benefit from compression if they get leg swelling with increased activity, even at 2 months. If patient has any specific pain (like trochanteric bursitis from overuse), PT might apply ultrasound or iontophoresis for that local issue, but this is case-dependent. | Remarks: Clearance for more activities often occurs around now, patient likely has a follow-up with their orthopedic surgeon around 8-10 weeks. The surgeon may clear them for driving (if not already), for gentle sports (cycling, swimming fully allowed, golf swings, etc.), and possibly sexual activity if not discussed earlier (often allowed when comfortable after 6 weeks). It’s important that the patient continues to observe any remaining restrictions the surgeon has (some surgeons say avoid high-impact running or heavy lifting until 12 weeks). The rehab plan should be adjusted to patient’s real-life context: if patient works a physical job and plans to return at 3 months, start simulating some work tasks under supervision (e.g. lifting a 20 lb box properly, practicing getting in/out of a work vehicle). Encourage endurance training to ensure they can handle full-day activities. Overall, acknowledge the patient’s hard work, at 8 weeks they have come a long way, with likely drastic improvements in pain and function from baseline. |
| **Week 9** | Ongoing Monitoring: App-based weekly assessments continue (pain, difficulty, compliance). By week 9, many patients are highly independent, so the app might reduce prompt frequency, but the patient can contact therapist via app messaging if any new issue arises. Therapist checks data once this week to ensure progression (e.g. are they increasing reps/weight as planned). | Transitional Phase: Transition from formal rehab exercises toward more integrative activities. Focus on symmetrical movement patterns, e.g. can the patient squat, climb stairs, and walk with symmetry and confidence. Increase speed and agility gradually for those who need it (e.g. able to change direction while walking without discomfort). Essentially preparing for full return to life or work by Week 12. | Exercises: The home program now shifts emphasis to maintenance and integration. Still 2–3 dedicated exercise sessions weekly focusing on strength and flexibility, but also encouraging the patient to incorporate exercises into daily routine (like doing heel raises while washing dishes, mini-squats during breaks, etc., to keep muscles active). For those aiming higher activity: add mild plyometric drills if appropriate (for example, side-to-side mini hops or stepping over low obstacles), only for fit patients and with therapist guidance, to gradually reintroduce impact. Continue strengthening with higher resistance if available (could move to gym equipment for leg press, hip abductor machine, etc., with guidance on safe use). Balance training can advance to dynamic challenges: e.g. gentle single-leg mini squats, or playing catch while standing on one leg (to simulate functional balance needs). Endurance: encourage variety, longer walks, maybe light hikes on easy trails, or swimming laps. By now, patient might easily tolerate 30+ minutes of continuous activity; aim for at least 150 minutes of moderate activity per week as a general health goal (as per public health guidelines). | Modalities: No routine modalities; the patient primarily uses active recovery (stretching, perhaps ice only after any unusually intense activity). They might use a heat pack if feeling stiff before an exercise session, but most find a regular warm-up suffices. Essentially, the focus is on active modalities (exercise) rather than passive now. | Remarks: This is a period where the patient may feel “almost normal.” It’s important to prevent any overconfidence injuries, counsel them to continue increasing activity gradually. For example, if returning to golf, don’t play 18 holes immediately, start with a half bucket of balls at the driving range. If returning to gym workouts, avoid high-impact aerobics or heavy weightlifting until after 3 months and cleared. Also discuss any work conditioning if they have a job that requires more physical effort: possibly simulate tasks or gradually resume duties part-time. Ensure they understand that even though pain is mostly gone, the biological healing of hip soft tissues continues through 3+ months, so they should still avoid extreme stress on the joint (no jumping from heights, no contact sports yet, etc.). Encourage them to keep using the good body mechanics and posture habits learned during rehab in their everyday life to protect their new hip. |
| **Week 10** | Follow-up (Remote): A brief phone call or video drop-in may be done around week 10 for those who had significant issues earlier, to verify they have resolved. Otherwise, the patient continues largely independently. The app might prompt a short survey this week asking if the patient has any concerns as they approach the end of formal rehab. | Confidence & Endurance: By week 10, the goal is for the patient to report feeling confident in their hip during most activities. They should be capable of extended walking (1+ mile) without rest, able to do a full flight of stairs up and down with no railing (if balance allows), and perform common tasks (lifting moderate items, getting on/off floor if needed) without difficulty. Preparing for self-managed fitness post-rehab. | Exercises: Emphasize functional training and any fine-tuning in these final weeks. For example, if patient still struggles with one-legged balance, add a specific drill like single-leg stance while doing arm movements (to mimic real-life balance challenges). If they have access to a gym, integrate machine exercises that they can continue post-rehab (leg press, hamstring curls, hip abductor machine) to solidify strength gains. Agility: introduce mild zig-zag or weave walking drills, practice quick turns while walking to simulate avoiding obstacles. Strength endurance: challenge with higher rep sets (e.g. 20 sit-to-stand in a row, carrying light weights while doing step-ups to simulate carrying groceries up stairs). Frequency can be down to 2–3 formal exercise days/week, as daily activities are now quite active. Encourage adding variety: maybe a yoga or Pilates session (with modifications as needed) for flexibility and core strength, or gentle low-impact aerobics class, easing them into regular fitness activities outside of “rehab.” | Modalities: No specific modalities; patient uses normal post-exercise strategies like any person would (cool-down stretches, maybe an ice pack if something is sore). | Remarks: At this point, many patients might be discharged from formal PT if they haven’t been already, with perhaps a final check scheduled at 12 weeks. It’s crucial to discuss the plan for after 12 weeks: the patient should continue an independent exercise routine to maintain and further improve their hip function. Provide them with a written home exercise maintenance program or transition them to community resources (like a gym program or arthritis exercise group). Remind them that the implant is strong, but the muscles around need continued conditioning, encourage lifelong activity. Also, check mental outlook: some patients at week 10 may feel they’ve plateaued; reassure them that strength and endurance will continue to improve for up to a year post-op with continued effort, and this last push in rehab will get them ready for that journey. |
| **Week 11** | Self-Monitoring: The system may prompt the patient to self-assess against initial benchmarks, for example, compare their current stair climbing ability or walking distance to how it was at the start. No formal check-in unless patient reaches out. Therapist prepares discharge summary in anticipation of next week’s final review. | Return to Recreation: Focus on any remaining specific goals (maybe playing a sport, dancing, or other personal goal). By now, patient’s strength and range should be at or near pre-surgery levels (or better, given pre-op arthritis pain). Work on any task that still feels challenging to the patient so that they feel ready to resume it fully after rehab. Endurance goal: be able to comfortably do at least 30 minutes of continuous moderate activity (which corresponds to being able to participate in typical recreational activities). | Exercises: Tapering formal exercises as the patient transitions to self-guided fitness. Ensure proper technique on all exercises has been mastered, patient should be doing them with excellent form now. Possibly simulate higher-level activities: if patient wants to jog eventually, maybe this week try a few jogging steps or light trot within parallel bars or on grass to gauge comfort (only if appropriate and cleared; many patients may choose not to run at all after THA, which is fine). If patient enjoyed a particular exercise, incorporate it into a sustainable routine (for instance, if they liked cycling, plan for cycling 2× week as cardio; if they liked certain PT exercises like bridges or clamshells, they can continue those a couple times a week for maintenance). Basically, finalize a well-rounded exercise routine that the patient can continue post rehab. | Modalities: None. By week 11, the patient manages any minor aches with occasional rest or over-the-counter methods. They should not require therapeutic modalities regularly. | Remarks: This is essentially a practice week for life after rehab. Patient should feel comfortable that they can keep improving even without constant therapist oversight. Discuss any final questions or fears the patient has about using their new hip fully. For example, some may worry about dislocation with certain moves, at this stage, if all has healed and they’ve been educated, they can generally resume nearly full movement (unless specific surgeon guidance otherwise). Reiterate long-term precautions (like avoiding extremely high-impact activities or being mindful of falls). If the patient has returned to work or is about to, make sure any needed ergonomic adjustments or strategies have been reviewed. Essentially, ensure the patient feels empowered and knowledgeable to manage their joint health moving forward. |
| **Week 12** | Final Evaluation (12-week outcome): In-person comprehensive assessment. Outcome measures repeated: HOOS Jr or other patient-reported outcome to quantify improvement from baseline, Timed Up and Go, 6-minute walk, stair test, etc., to document gains. Physical exam: check that hip ROM is near normative (often flexion 120°, extension 0°, rotation and abduction within functional range), muscle strength should be close to 5/5 in major groups. Gait and balance assessment to confirm normalization. Discuss if patient achieved their personal goals (e.g. “Can now walk around the block with no pain” etc.). App Data: final review of compliance data, typically by week 12, adherence has been high and correlates with better outcomes. | Outcomes & Discharge: Confirm that the patient can safely discontinue formal rehab. By week 12, goals are that the patient is independent in all ADLs and basic recreational activities, has minimal or no pain with routine activities, and understands how to continue exercise on their own. If any goals are unmet, create a plan (possibly an extension of therapy or referral if needed). Otherwise, celebrate success and provide guidance for maintaining improvements. | Exercises: After final testing, therapist may run the patient through a condensed version of their program as a warm-up and to ensure they have mastered it. No new exercises are introduced now, rather, the patient is given a long-term fitness plan. For example, continue strength training twice weekly (exercises like squats, lunges, side-lying leg lifts can be done lifelong), do aerobic exercise at least 3 times weekly (walking, cycling, swimming, etc.), and keep up with flexibility exercises (like hip flexor, hamstring stretches after workouts). The importance of cross-training is mentioned to avoid over-stressing the joint with one activity. Essentially, the “exercise” component this week is transitioning to unsupervised wellness activities. | Modalities: Not applicable. The focus is on self-maintenance now. If the patient ever has issues in the future (like a flare-up of pain after unusually heavy activity), they are advised on modalities (ice, rest) and to contact physician or PT if needed. | Remarks: Discharge Education: Provide detailed instructions on what to do after formal rehab ends. This includes recognizing any warning signs that should prompt medical follow-up (e.g. increasing pain, swelling, or any unusual clicking or instability in the hip, though rare at this stage). Encourage patient to attend routine follow-ups with surgeon (often at 3 months or 1 year post-op). Many patients at 12 weeks are eager to try more vigorous activities, give sport-specific guidance (for instance, okay to golf and swim; okay to cycle; caution with running or singles tennis until beyond 3–6 months and discuss with surgeon). Encourage weight management and healthy lifestyle to prolong the life of the implant. Finally, congratulate the patient on their progress: by 3 months post-THA, most have dramatically improved pain and function, and adherence to this 12-week program has been key in their successful recovery. |

**Adaptive Training Regulation Protocols**

**Baseline Tailoring**

From the program’s start, individual patient characteristics are used to adjust the training difficulty. Key baseline factors influencing the rehab plan include:

1. Preoperative Function and Strength: Patients enter rehab with varying ability levels. Those with low baseline mobility or strength (for example, very limited walking ability or weak quadriceps before surgery) begin at a gentler intensity. The system might start them with simpler exercises (more assisted range-of-motion, lower resistance) and a slower progression timeline. Conversely, patients who were fit and active pre-surgery can safely handle more challenging exercises sooner, their program might start at a slightly higher difficulty and progress faster. Baseline functional tests (such as a Timed Up and Go or 6-minute walk done at initial eval) guide these decisions: a slower TUG time or poor balance suggests the need for a more cautious start, whereas a faster TUG indicates readiness for more advanced tasks earlier.
2. Age and Comorbidities: Older patients or those with multiple health issues (e.g. diabetes, cardiac conditions) are regulated to a more conservative program initially. The system may build in extra rest days or lower target repetitions to accommodate potentially slower tissue healing and lower exercise tolerance. Balance training is emphasized earlier for older patients to reduce fall risk. In contrast, younger patients often can progress more quickly and may benefit from higher-level strengthening and endurance challenges to meet their lifestyle needs. For example, a healthy 50-year-old might advance to single-leg exercises by week 6, whereas an 80-year-old might stick to bilateral support exercises a bit longer. Comorbid conditions (like significant arthritis in the other leg or a history of back problems) are also considered, the program will modify exercises to avoid exacerbating those issues (for instance, someone with back pain might do more core stabilization and avoid certain bending exercises initially).
3. Body Mass and Build: Patients with higher BMI (overweight/obesity) often experience more stress on the new hip and may have slightly slower progress. The training is regulated to be joint-friendly, low-impact exercises (aquatic therapy, cycling) are emphasized, and impact or high-force activities are introduced very cautiously. The therapist and system also watch for any signs of knee or back strain (common if weight adds load) and adjust accordingly (perhaps keeping squats shallower or using assistive devices longer to unload weight). A lighter-weight or very muscular patient might tolerate quicker progression in resistance exercises.
4. Surgical Approach and Precautions: Baseline surgical factors alter the training. For example, a patient with a posterior approach THA has strict precautions (no deep bending, no internal rotation/adduction initially), so the system automatically omits any exercise violating those ranges in early weeks and gradually introduces them only after the precaution period (typically after 6–8 weeks, per surgeon’s clearance). An anterior approach patient might have more caution with hip extension and external rotation early on, so exercises like prone hip extensions are delayed. The app’s program is customized at baseline to reflect these precautions, ensuring safety by design and adapting the range-of-motion targets accordingly.
5. Baseline Pain and Confidence: Some patients start rehab with high pain levels or fear of movement. The program identifies these via initial pain scores and psychological questionnaires (if available). A patient with severe pain or low confidence is given a gentler start: more reassurance and pain-control modalities (like extra icing, relaxation techniques), and very small exercise increments to build trust. Alternatively, a patient reporting minimal pain and high motivation at baseline can progress through exercises at a standard or accelerated pace, as they are less inhibited. The system’s AI might tag a high-pain individual for closer monitoring and more frequent check-ins, whereas a low-pain individual might get motivational challenges to push themselves a bit more.

**Ongoing Progress Adjustments**

Throughout the 12-week program, the physiotherapist and digital system continuously regulate the training intensity based on the patient’s recovery progress:

1. Weekly App Assessments: Each week, patients input pain scores and exercise difficulty ratings. The wearable sensors (for those in the sensor-feedback group) also provide objective data like range of motion achieved and number of repetitions completed. The system uses these inputs to auto-adjust the upcoming week’s program. For example, if a patient consistently reports exercises are “too easy” with no pain increase, the app will suggest advancing the level, perhaps increasing reps by 10–20% or introducing a harder exercise a bit earlier than scheduled. The therapist reviews these suggestions and approves or fine-tunes them. On the other hand, if a patient’s pain spikes or they struggle to complete sessions, the system flags this. The therapist may then regress the program temporarily: reducing repetition count, adding an extra rest day, or substituting an easier exercise (e.g. swapping a full squat for a higher sit-to-stand) until the patient can comfortably progress again. This dynamic modulation ensures the patient isn’t overtaxed, while still challenging them enough to improve.
2. Monthly In-Person Evaluations: At roughly weeks 4 and 8, formal assessments guide larger program adjustments. Suppose at Week 4 evaluation the patient has quad weakness still limiting their stair climbing, the therapist will modify the next phase to include more targeted quadriceps strengthening (like adding weighted step-ups or neuromuscular electric stimulation in clinic) and perhaps keep the patient on the current level of balance exercises a bit longer until strength improves. If at Week 8 the patient shows near-full recovery and easily meets discharge criteria early, the therapist can accelerate the final phase: introducing advanced functional drills or even considering ending supervised rehab a bit sooner with a transition to unsupervised exercise. In contrast, if at 8 weeks the patient lags behind (say they still have a moderate limp or ROM deficit), the training protocol can be extended or intensified in specific areas, the therapist might schedule a couple of extra in-person sessions focusing on gait training, and the app will continue structured exercises beyond week 12 if needed (the program can be extended for a few weeks for those who need more time).
3. Data-Driven Alerts: The wearable sensor system provides real-time alerts for concerning trends. For instance, if a patient’s activity levels drop significantly for a few days (possibly indicating a setback or low adherence), the system notifies the therapist. In response, the therapist contacts the patient to troubleshoot, maybe the patient had increased pain, in which case the therapist will adjust the program (reducing difficulty) and advise on pain management. If the patient was just feeling unmotivated, the therapist provides encouragement or resets goals to get them back on track. Another scenario: the sensor detects abnormal movement patterns, such as the patient not achieving the expected range on an exercise for several sessions in a row. This could mean a new stiffness or fear has arisen. The therapist would then intervene, perhaps scheduling a video session to observe the exercise form and then modifying the exercise (or providing additional manual therapy if available) to address the issue.
4. Criteria-Based Progression: The program has built-in criteria that the patient should meet before advancing to certain high-level exercises. For example, the patient must be able to perform 10 straight leg raises with good form and no lag before progressing to resisted leg raises or standing hip swings. Likewise, to begin single-leg balance exercises, they should demonstrate they can stand on the operative leg for, say, 10 seconds with support nearby. These criteria ensure safety. The system checks the patient’s performance (via sensor or self-report) and only unlocks the next difficulty when the patient consistently meets the benchmarks. If benchmarks aren’t met, the system keeps the patient at the current level a bit longer and might introduce remedial exercises. For instance, if the patient cannot do a single-leg stand yet, the program will automatically add more hip abductor strengthening and supported balance exercises rather than moving on.
5. Therapist Oversight and Modification: The physiotherapist remains the ultimate decision-maker for progression. They review progress dashboards at least weekly. If a patient is exceeding goals, the therapist might choose to skip ahead in the protocol, for example, introduce light jogging at week 10 instead of 12 for a very athletic patient who has no pain and excellent strength. In contrast, if a patient is struggling (perhaps due to an intervening issue like a back flare-up or opposite knee pain), the therapist can modify the protocol to be more gentle for a period. They might focus on cross-training (like more pool work) for a week to maintain fitness while letting an irritated joint calm down, then resume the hip exercises. The therapist documents these changes, and the system adjusts the scheduled progression accordingly.
6. Patient Feedback and Goals: The program also considers the patient’s own goals and subjective feedback. If a patient feels an exercise is not challenging or conversely is too fearful of a certain movement, the therapist can substitute alternative exercises that achieve the same purpose. For example, if a patient is afraid of doing lateral stepping due to fear of falling, the therapist might employ parallel bar side-walks or use a harness in clinic (if available) to build confidence, then reintroduce it later. The motivation system in the app allows patients to set personal goals (like “walk around the park with my grandchild by week 8”); if such a goal is not on track, the therapist will adjust training to target that functionality (maybe adding more walking endurance drills). This patient-centered adjustment ensures the rehab remains relevant and appropriately challenging, not just following a rigid template.

In summary, the regulation protocol is highly responsive: starting from an individualized baseline plan based on each patient’s characteristics, and continuously fine-tuning the exercise intensity and content in response to performance and recovery status. This adaptive approach, supported by real-time data and therapist expertise, helps each patient progress at an optimal pace. Faster recoverers are safely challenged to reach higher levels of function, while those needing more time receive a tempered program that prioritizes safety and steady improvement. By the end of 12 weeks, thanks to this regulated approach, each patient achieves the best possible outcome for their situation, having neither under-trained nor over-stressed their new hip.

**References**

Konnyu KJ, Pinto D, Cao W, et al. Rehabilitation for total hip arthroplasty: a systematic review. *Am J Phys Med Rehabil.* 2023;102(1):11-18.
