# Supplementary material for: The Efficiency and Cost-Effectiveness of Wearable Sensors in a Digital Physiotherapeutic Total Hip Arthroplasty–Specific Training System for Patients After Total Hip Arthroplasty: Randomized Controlled Trial
Source: JMIR Mhealth Uhealth. 2026 Jul 21;14:e93050. doi: 10.2196/93050 (PMC13387417; doi:10.2196/93050)
Supplement: Multimedia Appendix 2 [file mhealth-v14-e93050-s002.docx]

Table S1. Baseline characteristics of the IMU group and control group (PP population)

|  | **Sample characteristic** | **IMU**  **(N=106)** | **Control group (N=104)** | **P value** |
| --- | --- | --- | --- | --- |
| **Demographics** | Male Patients (no. [%]) | 54 (50.94) | 51 (49.04) | 0.783 |
|  | Age (yr) | 69 (5.28) | 69 (5.06) | 0.913 |
|  | Body mass index (kg/m^2^) | 22 (2.02) | 22 (1.86) | 0.392 |
|  | Occupation (no. [%]) |  |  | 0.713 |
|  | Manual worker | 74 (69.81) | 75 (72.12) |  |
|  | Non-manual worker | 32 (30.19) | 29 (27.88) |  |
|  | Education level (no.[%]) |  |  | 0.811 |
|  | Lower than high school | 80 (75.47) | 77 (74.04) |  |
|  | Equal/higher to high school | 26 (24.53) | 27 (25.96) |  |
|  | Insurance type (no. [%]) |  |  | 0.148 |
|  | Government | 73 (68.87) | 67 (64.42) |  |
|  | Commercial | 9 (8.49) | 18 (17.31) |  |
|  | Self-financed | 24 (22.64) | 19 (18.27) |  |
|  | Current Smoker (no. [%]) | 41 (38.68) | 40 (38.46) | 0.974 |
|  | Current Alcohol use (no. [%]) | 29 (27.36) | 27 (25.96) | 0.819 |
|  | Paracetamol and NSAIDs (no. [%]) | 16 (15.09) | 18 (17.31) | 0.663 |
|  | Side of Hip (Left no. [%]) | 64 (60.38) | 61 (58.65) | 0.799 |
|  | Living alone (no. [%]) | 69 (65.09) | 65 (62.50) | 0.696 |
| **Function** | HOOS-pain | 41 (4.24) | 41 (4.90) | 0.862 |
|  | HOOS-symptoms | 40 (5.47) | 40 (5.42) | 0.698 |
|  | HOOS-function in ADL | 41 (4.80) | 41 (4.40) | 0.805 |
|  | HOOS-sports and recreation | 25 (3.15) | 25 (3.55) | 0.903 |
|  | HOOS-hip related quality of life | 25 (3.56) | 25 (3.64) | 0.730 |
|  | HOOS-Overall | 38 (2.58) | 38 (2.43) | 0.877 |
|  | TUG (seconds) | 31 (4.56) | 30 (4.88) | 0.466 |
|  | Berg balance test | 35 (3.56) | 35 (3.92) | 0.898 |
| **PROMs** | SF36-physical component summary | 25 (5.12) | 25 (4.70) | 0.941 |
|  | SF36 Mental component summary | 40 (5.59) | 40 (6.71) | 0.971 |
|  | HADS Anxiety subscale (HAD-A) | 10 (2.10) | 10 (2.04) | 0.951 |
|  | HADS Depression subscale (HAD-D) | 10 (1.89) | 10 (2.12) | 0.962 |

IMU: Inertial Measurement Unit; PP: per-protocol; NSAIDs: Nonsteroidal Anti-Inflammatory Drugs; HOOS: Hip Disability and Osteoarthritis Outcome Score; ADL: Activities of Daily Living; TUG: Timed Up and Go test; PROMS: patient-reported outcome measures; 36-Item Short Form Health Survey; HADS: Hospital Anxiety and Depression Scale.

Table S2 Changes in outcomes for the IMU and control groups at 6-week, 12-week and 24-week follow-up, PP analysis

| **Outcome** | **6-week post-surgery** | | | **12-week post-surgery** | | | **24-week post-surgery** | | |
| --- | --- | --- | --- | --- | --- | --- | --- | --- | --- |
|  | **IMU**  **(N=106)** | **Control group (N=104)** | **P value** | **IMU**  **(N=106)** | **Control group (N=104)** | **P value** | **IMU**  **(N=106)** | **Control group (N=104)** | **P value** |
| **Function Outcomes** |  |  |  |  |  |  |  |  |  |
| HOOS-pain | 13.30 (8.16) | 8.47 (4.96) | 0.000 | 23.61 (9.41) | 23.45 (9.36) | 0.898 | 32.55 (10.23) | 32.64 (10.38) | 0.946 |
| HOOS-symptoms | 17.19 (8.41) | 12.14 (7.33) | 0.000 | 29.10 (10.74) | 28.94 (12.75) | 0.921 | 37.46 (11.71) | 37.84 (13.05) | 0.827 |
| HOOS-function in ADL | 15.94 (8.18) | 13.15 (5.93) | 0.005 | 29.17 (10.44) | 26.72 (9.42) | 0.075 | 35.45 (10.99) | 35.05 (10.07) | 0.781 |
| HOOS-sports and recreation | 21.98 (10.71) | 15.15 (9.53) | 0.000 | 40.77 (16.05) | 40.35 (12.03) | 0.831 | 51.63 (17.81) | 51.45 (12.34) | 0.932 |
| HOOS-hip related quality of life | 21.38 (11.38) | 17.38 (7.04) | 0.003 | 42.08 (16.36) | 41.89 (13.73) | 0.929 | 54.92 (15.29) | 54.92 (14.69) | 0.999 |
| HOOS-Overall | 16.57 (5.17) | 12.51 (3.02) | 0.000 | 30.20 (5.89) | 29.08 (5.18) | 0.145 | 38.53 (6.12) | 38.44 (5.94) | 0.918 |
| TUG (seconds) | -6.23 (3.74) | -3.65 (3.06) | 0.000 | -8.92 (4.15) | -7.63 (3.86) | 0.021 | -11.38 (4.38) | -10.94 (4.60) | 0.483 |
| Berg balance test | 5.32 (2.42) | 4.42 (1.96) | 0.004 | 7.60 (2.70) | 7.12 (2.28) | 0.158 | 10.27 (2.74) | 10.29 (2.58) | 0.968 |
| **PROMs** |  |  |  |  |  |  |  |  |  |
| SF36-physical component summary | 29.95 (2.72) | 22.55 (2.51) | 0.000 | 42.45 (3.67) | 42.31 (3.75) | 0.777 | 50.14 (4.70) | 50.14 (4.38) | 0.997 |
| SF36 Mental component summary | 20.11 (2.10) | 13.69 (2.14) | 0.000 | 25.17 (4.45) | 25.15 (3.51) | 0.977 | 33.17 (4.45) | 33.15 (3.51) | 0.977 |
| HADS Anxiety subscale (HAD-A) | -2.58 (0.50) | -1.47 (0.50) | 0.000 | -3.40 (1.53) | -3.29 (0.83) | 0.528 | -4.40 (1.53) | -4.25 (0.93) | 0.405 |
| HADS Depression subscale (HAD-D) | -2.48 (0.50) | -1.44 (0.50) | 0.000 | -3.56 (1.30) | -3.48 (1.13) | 0.653 | -4.56 (1.30) | -4.48 (1.13) | 0.653 |
| **Patients’ evaluation on current intervention*** |  |  |  |  |  |  |  |  |  |
| Convenience | 0.00 (0.24) | 0.00 (0.28) | 1.000 | 4.97 (0.17) | 0.01 (0.26) | 0.000 | 0.00 (0.24) | 0.00 (0.28) | 1.000 |
| Ease of use/access | 0.01 (0.22) | -0.01 (0.26) | 0.566 | 4.97 (0.17) | 0.00 (0.28) | 0.000 | 0.01 (0.26) | -0.01 (0.30) | 0.619 |
| Helpfulness | -0.01 (0.22) | -0.01 (0.26) | 0.996 | 4.97 (0.17) | -0.01 (0.26) | 0.000 | 0.00 (0.28) | -0.01 (0.26) | 0.795 |
| Communication with the care provider | 0.00 (0.24) | 0.00 (0.28) | 1.000 | 4.96 (0.19) | 0.01 (0.26) | 0.000 | 0.00 (0.24) | 0.01 (0.26) | 0.781 |

IMU: Inertial Measurement Unit; PP: per-protocol; NSAIDs: Nonsteroidal Anti-Inflammatory Drugs; HOOS: Hip Disability and Osteoarthritis Outcome Score; ADL: Activities of Daily Living; TUG: Timed Up and Go test; PROdwMS: patient-reported outcome measures; 36-Item Short Form Health Survey; HADS: Hospital Anxiety and Depression Scale.

Values represent the mean change from baseline for each group, reported as mean (standard deviation), with P values comparing between-group differences at each follow-up point. Functional outcomes include primary endpoints and secondary performance-based tests

Table S3 Effectiveness estimates from linear mixed effects models for the IMU and control groups at 6-week, 12-week and 24-week follow-up, PP analysis

| **Outcome** | **6-week post-surgery** | | | **12-week post-surgery** | | | **24-week post-surgery** | | |
| --- | --- | --- | --- | --- | --- | --- | --- | --- | --- |
|  | **Coefficient** | **95% CI** | **P value** | **Coefficient** | **95% CI** | **P value** | **Coefficient** | **95% CI** | **P value** |
| **Function Outcomes** |  |  |  |  |  |  |  |  |  |
| HOOS-pain | 2.527 | (1.655, 3.399) | 0.000 | 1.781 | (0.545, 3.017) | 0.005 | 1.351 | (-0.136, 2.839) | 0.075 |
| HOOS-symptoms | 2.327 | (1.272, 3.382) | 0.000 | 1.576 | (0.002, 3.149) | 0.050 | 1.065 | (-0.833, 2.963) | 0.271 |
| HOOS-function in ADL | 1.326 | (0.379, 2.273) | 0.006 | 1.648 | (0.230, 3.066) | 0.023 | 1.286 | (-0.420, 2.993) | 0.140 |
| HOOS-sports and recreation | 3.224 | (1.836, 4.612) | 0.000 | 2.073 | (0.134, 4.012) | 0.036 | 1.435 | (-0.887, 3.758) | 0.226 |
| HOOS-hip related quality of life | 2.138 | (0.942, 3.334) | 0.000 | 1.616 | (-0.270, 3.502) | 0.093 | 1.293 | (-0.959, 3.545) | 0.261 |
| HOOS-Overall | 2.022 | (1.460, 2.584) | 0.000 | 1.714 | (0.910, 2.518) | 0.000 | 1.292 | (0.324, 2.260) | 0.009 |
| TUG (seconds) | -1.173 | (-1.611, -0.734) | 0.000 | -1.122 | (-1.738, -0.506) | 0.000 | -0.873 | (-1.582, -0.164) | 0.016 |
| Berg balance test | 0.460 | (0.184, 0.736) | 0.001 | 0.490 | (0.111, 0.869) | 0.011 | 0.372 | (-0.058, 0.803) | 0.090 |
| **PROMs** |  |  |  |  |  |  |  |  |  |
| SF36-physical component summary | 3.720 | (3.382, 4.059) | 0.000 | 2.531 | (2.012, 3.049) | 0.000 | 1.882 | (1.224, 2.540) | 0.000 |
| SF36 Mental component summary | 3.232 | (2.953, 3.510) | 0.000 | 2.198 | (1.773, 2.622) | 0.000 | 1.681 | (1.130, 2.232) | 0.000 |
| HADS Anxiety subscale (HAD-A) | -0.547 | (-0.611, -0.484) | 0.000 | -0.412 | (-0.520, -0.305) | 0.000 | -0.355 | (-0.498, -0.212) | 0.000 |
| HADS Depression subscale (HAD-D) | -0.497 | (-0.560, -0.434) | 0.000 | -0.357 | (-0.466, -0.248) | 0.000 | -0.287 | (-0.432, -0.142) | 0.000 |
| **Patients’ evaluation on current intervention*** |  |  |  |  |  |  |  |  |  |
| Convenience | -0.003 | (-0.027, 0.022) | 0.840 | -0.001 | (-0.023, 0.020) | 0.923 | -0.002 | (-0.021, 0.017) | 0.858 |
| Ease of use/access | 0.010 | (-0.013, 0.033) | 0.387 | 0.015 | (-0.006, 0.036) | 0.170 | 0.016 | (-0.004, 0.036) | 0.111 |
| Helpfulness | -0.007 | (-0.032, 0.019) | 0.609 | -0.001 | (-0.023, 0.021) | 0.950 | -0.002 | (-0.023, 0.019) | 0.863 |
| Communication with the care provider | 0.008 | (-0.014, 0.031) | 0.479 | 0.000 | (-0.021, 0.021) | 0.978 | 0.000 | (-0.019, 0.020) | 0.960 |

IMU: Inertial Measurement Unit; PP: per-protocol; NSAIDs: Nonsteroidal Anti-Inflammatory Drugs; HOOS: Hip Disability and Osteoarthritis Outcome Score; ADL: Activities of Daily Living; TUG: Timed Up and Go test; PROMS: patient-reported outcome measures; 36-Item Short Form Health Survey; HADS: Hospital Anxiety and Depression Scale.

Each coefficient represents the estimated between-group difference in the change from baseline (IMU group minus Control group) for the specified outcome at that follow-up time point. Positive coefficients indicate higher scores in the IMU group compared to the Control group, whereas negative values indicate lower scores in the IMU group. Each estimate is presented with its 95% confidence interval (CI) and corresponding *P* value. All outcome measures were adjusted for baseline values in the model.

Table S4 Average total cost per patient in the IMU group and control group during the 24 weeks after the surgery, PP analysis

| **Cost category (CNY)** | **IMU (N=106)** | **Control group (N=104)** | **P value** |
| --- | --- | --- | --- |
| **Medical Cost** |  |  |  |
| Cost during hospital (include hospital stay, surgery fee, medication, implants during surgery) | 69619.18 (5393.02) | 70568.43 (5006.07) | 0.000 |
| Physical therapist cost (include instrument) | 6980.00 (0.00) | 8413.79 (1049.09) | 0.188 |
| Primary care cost | 250.94 (37.78) | 1497.12 (186.67) | 0.000 |
| **Non-medical cost** |  |  |  |
| Paid home cost | 1178.32 (366.96) | 1455.68 (360.80) | 0.000 |
| Transportation cost | 799.81 (137.93) | 1975.03 (261.42) | 0.000 |
| Nutrition cost | 4502.31 (481.50) | 5093.35 (510.32) | 0.000 |
| **Opportunity Cost** |  |  |  |
| Lost wages for patients | 1675.24 (7150.41) | 1646.52 (6201.70) | 0.000 |
| Lost wages for families | 3265.58 (4512.66) | 3687.64 (4709.77) | 0.975 |
| **TOTAL COST** | 87967.32 (8854.19) | 94396.30 (10037.74) | 0.508 |

PP: per-protocol; CNY: Chinese Yuan; IMU: Inertial Measurement Unit.

Costs are categorized into medical costs (e.g., costs of hospital stay, surgery, medication used during hospital stay, ICU if need, and costs of the physiotherapist or digital platform use, and any hospital stay related to the rehabilitation, cost of primary care visit), non-medical costs (e.g., transportation expenses for clinic visits and nutritional supplements), and opportunity costs (productivity losses such as lost wages for patients and family caregivers). Values are reported as mean cost per patient in each category for the 24-week period, with P values for the between-group comparisons.

Table S5 Incremental cost-effectiveness ratio (ICER), PP analysis

| **Items** | **Main analysis - mixed effects** |
| --- | --- |
| Incremental cost, CNY | -6066.17 |
| Incremental HOOS-pain | 1.351 (-0.136, 2.839) |
| Incremental HOOS-symptoms | 1.065 (-0.833, 2.963) |
| Incremental HOOS-function in ADL | 1.286 (-0.420, 2.993) |
| Incremental HOOS-sports and recreation | 1.435 (-0.887, 3.758) |
| Incremental HOOS-hip related quality of life | 1.293 (-0.959, 3.545) |
| Incremental HOOS-Overall | 1.292 (0.324, 2.260) |
| Incremental TUG (seconds) | -0.873 (-1.582, -0.164) |
| Incremental Berg balance test | 0.372 (-0.058, 0.803) |
| Incremental SF36-physical component summary | 1.882 (1.224, 2.540) |
| Incremental SF36 Mental component summary | 1.681 (1.130, 2.232) |
| Incremental HADS Anxiety subscale (HAD-A) | -0.355 (-0.498, -0.212) |
| Incremental HADS Depression subscale (HAD-D) | -0.287 (-0.432, -0.142) |
| Incremental Convenience | -0.002 (-0.021, 0.017) |
| Incremental Ease of use/access | 0.016 (-0.004, 0.036) |
| Incremental Helpfulness | -0.002 (-0.023, 0.019) |
| Incremental Communication with the care provider | 0.000 (-0.019, 0.020) |
| **Incremental cost-effectiveness ratio** |  |
| ICER HOOS-pain | -4488.64 |
| ICER HOOS-symptoms | -5696.41 |
| ICER HOOS-function in ADL | -4715.66 |
| ICER HOOS-sports and recreation | -4226.04 |
| ICER HOOS-hip related quality of life | -4692.57 |
| ICER HOOS-Overall | -4695.24 |
| ICER TUG (seconds) | 6951.89 |
| ICER Berg balance test | -16286.04 |
| ICER SF36-physical component summary | -3223.14 |
| ICER SF36 Mental component summary | -3609.33 |
| ICER HADS Anxiety subscale (HAD-A) | 17099.41 |
| ICER HADS Depression subscale (HAD-D) | 21127.49 |
| ICER Convenience | 3469951.95 |
| ICER Ease of use/access | -374110.85 |
| ICER Helpfulness | 3265947.02 |
| ICER Communication with the care provider | -12215404.75 |

IMU: Inertial Measurement Unit; ITT: intention-to-treat; NSAIDs: Nonsteroidal Anti-Inflammatory Drugs; HOOS: Hip Disability and Osteoarthritis Outcome Score; ADL: Activities of Daily Living; TUG: Timed Up and Go test; PROMS: patient-reported outcome measures; 36-Item Short Form Health Survey; HADS: Hospital Anxiety and Depression Scale.

The table displays the incremental cost, incremental effect in primary outcome scores, and the resulting incremental cost-effectiveness ratio (ICER). ICER is defined as the incremental cost divided by the incremental effect, representing the additional cost per unit improvement in outcome for the IMY group versus the Control group. Incremental effects are given for primary outcome measures. ICER values are presented for the 24-week follow-up under both the intention-to-treat (with multiple imputation) and per-protocol analyses. A negative ICER indicates that the IMU intervention achieved equivalent or better outcomes at a lower cost compared to Control.

Table S6 Patients’ adherence to treatment (ITT population)

| **Outcome measure*** | **IMU**  **(N=120)** | **Control group (N=120)** | **P value** |
| --- | --- | --- | --- |
| Number of prescribed exercise sessions completed, mean ± SD (maximum to 60) | 51.6 (9.3) | 48.7 (10.6) | 0.002 |
| Number of weekly app-based assessments attended, mean ± SD (maximum to 12) | 10.5 (1.5) | 9.5 (1.8) | 0.000 |
| Number of scheduled weekly follow-up phone calls successfully completed, mean ± SD (maximum to 12) | 10.8 (1.6) | 9.3 (1.8) | 0.000 |

IMU: Inertial Measurement Unit; ITT: intention-to-treat;

*The patient adherence data were obtained from the Joymotion app, and the number of scheduled weekly follow-up phone calls was recorded and calculated by the research team.

Table S7 Adverse Events and Serious Adverse Events (ITT population)

| **Adverse events** | **IMU**  **(N=120)** | **Control group (N=120)** |
| --- | --- | --- |
| Patients with adverse events (no. [%]) | 7 (5.8) | 9 (7.5) |
| Events related to study therapy (no.) | 9 | 12 |
| Events unrelated to study therapy (no.) | 3 | 2 |
| Type of event (no.) |  |  |
| Involved hip |  |  |
| Pain | 4 | 5 |
| Swelling | 1 | 0 |
| Signs of superficial infection (swelling, redness, heat, or pus) | 1 | 1 |
| Other |  |  |
| Fall with minor symptoms | 2 | 2 |
| Nausea and dizziness | 1 | 0 |
| Low Back pain | 2 | 2 |
| Anxiety about knee recovery | 1 | 4 |
| **Serious adverse events*** |  |  |
| Patients with serious adverse events (no. [%]) | 3 (2.5) | 6 (5) |
| Events related to study therapy (no.) | 0 | 0 |
| Events unrelated to study therapy (no.) | 3 | 6 |
| Type of event (no.) |  |  |
| Death | 0 | 1 |
| Hospitalization# | 2 | 2 |
| Prosthetic dislocation## | 0 | 1 |
| Degradation of the general condition | 0 | 1 |
| Unplanned surgery# | 1 | 1 |

IMU: Inertial Measurement Unit; ITT: intention-to-treat

*Patients with serious adverse events were automatically withdrawn from the study.

#The patients noted ‘Hospitalization’ included patients performed unplanned surgery

##One patient in control sustained prosthetic dislocation during her walking to the supermarket, and reduction was achieved by close procedure.
